# Increasing usable reads in RNA-seq protocols

**DOI:** 10.1016/j.isci.2026.116984

**Published:** 2026-07-30

**Authors:** Felix Pförtner, Eva Briem, Wolfgang Enard, Daniel Richter

**Affiliations:** 1 Anthropology and Human Genomics, Faculty of Biology, Ludwig-Maximilians Universität in Munich, Großhaderner Str. 2, Planegg-Martinsried 82152, Germany

**Keywords:** RNA-seq, transcriptomics, off-target priming, antisense reads, benchmarking, next generation sequencing

## Abstract

Bulk and single-cell RNA-seq are powerful tools for transcriptomic analysis, providing insights into molecular and cellular phenotypes. Costs constrain the biological insights obtainable within a given budget, and as sequencing prices decline, efficient library protocols have become a decisive factor. In this study, we introduce an approach to systematically optimize the number of usable reads generated by RNA-seq protocols. We applied this “funnel strategy” to prime-seq, an early-barcoding bulk RNA-seq protocol, by systematically testing critical protocol steps totaling 1,256 samples in 65 libraries. This resulted in the optimized prime-seq2 protocol that increases the number of usable reads by 60% and improves one of the most cost-efficient bulk RNA-seq protocols available. Our study also suggests that monitoring usable reads can serve as a valuable quality control for many RNA-seq protocols and sheds light on the complex interplay of experimental conditions that shape RNA-seq library composition and their interpretation.

## Highlights

• The read-funnel strategy pinpoints bottlenecks to maximize usable RNA-seq reads
• The systematic optimization increases usable reads by 60% for prime-seq2
• Usable and antisense reads increase statistical power without added sequencing costs

## Graphical abstract

**Figure:**
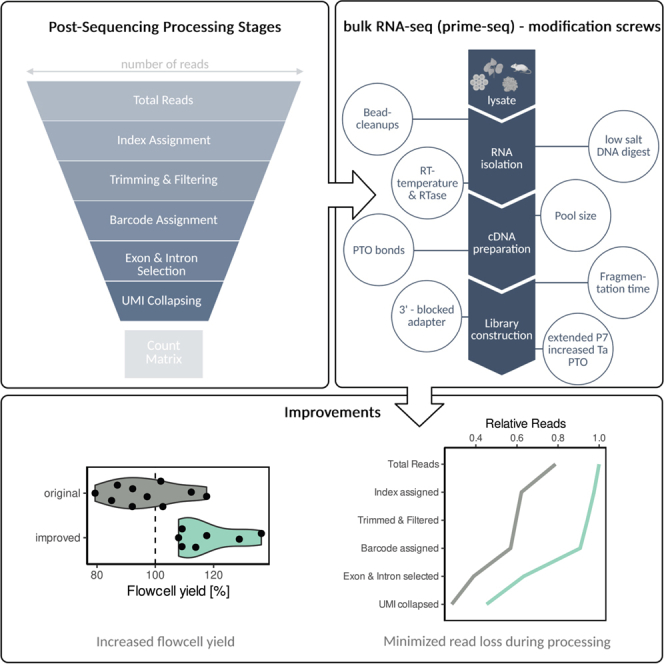

## Teaser

Genomics; Molecular biology; Biotechnology; Transcriptomics

## Introduction

RNA sequencing (RNA-seq) has revolutionized molecular biology by enabling comprehensive analyses of transcriptomes.^1^ This includes developments for genome annotation and isoform detection that were boosted by long-read sequencing technologies^2^ and comprehensive characterization of cell types, enabled by single-cell RNA-seq and spatial transcriptomics.^3^ While these exciting developments have justifiably garnered much attention, bulk RNA-seq, in which average expression levels of many cells are measured, remains an essential and complementary tool. Bulk RNA-seq is valued especially for its cost-efficiency and its easy and unbiased sampling procedure. This is of particular advantage when the cellular composition of samples is sufficiently homogeneous. Notably, the increasing availability of single-cell RNA-seq data, together with recent advances in deconvolution methods,^4^^,^^5^^,^^6^ is poised to significantly improve the interpretation—and thus the utility—of bulk RNA-seq data in the future.

Many different bulk RNA-seq protocols have been developed.^7^^,^^8^ They differ in aspects such as the type of RNAs (e.g., polyadenylated RNAs, short RNAs, rRNA-depleted RNAs, newly synthesized RNAs) and the parts of RNAs (5′ ends, 3′ ends, full-length) that get incorporated in sequencing libraries. They differ in their sensitivity, i.e., how many cells are needed as input; their accuracy, i.e., how well cDNA molecules in the library correlate with RNA abundance; their complexity, i.e., how many different genes are detected at a given sequencing depth; and their technical noise, i.e., the variation with which the detected RNAs are measured in technical replicates. Obviously, different questions and samples require different protocols. Here, we focus on probably the most frequent application of bulk RNA-seq, in which genome-wide expression levels of polyadenylated RNAs are correlated with variables of interest, such as drug treatment, genetic variants, or disease states. Data are usually interpreted by identifying differentially expressed genes and their enrichment in molecular pathways and biological processes. This type of bulk RNA-seq application has been called RNA-seq for differential gene expression,^1^ mRNA Sequencing^9^ or quantitative RNA-seq.^10^ It is conceptually similar to a quantitative reverse transcription polymerase chain reaction (reverse transcription (RT)-qPCR) for many genes and is becoming as routine, as important, and as affordable as RT-qPCR.^11^

As with many methods, costs are a crucial factor, as they limit the amount of biological insight within a given budget. Costs not only constrain the number of replicates and hence the statistical power to detect expression differences,^12^^,^^13^^,^^14^ but they also limit the number of variables (e.g., tissues, time points, treatments) that can be investigated. For RNA-seq, sequencing costs have long limited the number of samples to the notorious *N* = 3 per condition. Ten years ago, that could be justified by budget limitations when costs for generating and sequencing a bulk RNA-seq sample were maybe around $200. But sequencing costs have dropped 10-fold in the last ten years,^15^ and one million reads can cost less than one dollar on the latest illumina NovaSeq X machines. Hence, costs for sequencing an RNA-seq sample fell to $10–20, making costs of $60–164 per sample for commercial library generation kits the dominant cost factor.^16^ As a consequence, improvements such as miniaturization and early barcoding, originally developed to improve scRNA-seq cost-efficiency,^17^ have become increasingly important to leverage the full potential of bulk RNA-seq.

Early barcoding protocols (also called Tag-Seq, 3′ RNA-seq, or QuantSeq), in which a sample-specific barcode is introduced either at the 5′ or at the 3′ end of the polyT primed cDNA, allow for pooling cDNA before amplification and library generation. This decreases library reagent costs for non-commercial protocols to $2.53, $4.05, or $6.58 for prime-seq,^16^ BRB-seq,^11^ or DECODE-seq,^18^ respectively. In addition to the reagent costs per library, other factors such as necessary equipment, hands-on time, or failure rates contribute to cost-efficiency. Of note, the low costs per library of these protocols make kit-based RNA isolations of ∼$5 per sample a relevant factor, and protocols have established different methods to incorporate direct lysis or RNA purification steps.^16^^,^^19^^,^^20^^,^^21^ Importantly, costs are directly comparable only at the same efficiency, i.e., at the same statistical power to detect differently expressed genes.^22^ At a given number of replicates and sequencing reads, this depends mainly on the library complexity and the technical noise of the protocol.^23^^,^^24^ While comprehensive benchmarking for early barcoding protocols has not been done, they generally perform fairly similarly to each other and fairly similarly to commercial gold standards such as TruSeq with respect to technical noise and complexity.^11^^,^^16^ A potential drawback of early barcoding protocols is that mainly 5′ or 3′ ends of cDNAs are sequenced, and hence that expression levels are quantified per gene and not per transcript isoform. But as the available functional annotation is almost always gene-specific and not isoform-specific, most projects interpret the RNA-seq data anyway only at the level of differentially expressed genes. Additionally, quantifying expression levels of isoforms requires either long-read sequencing or deep short-read sequencing, which increases the sequencing costs per library considerably. Hence, the most cost-efficient way to quantify gene expression levels by RNA-seq is currently early barcoding RNA-seq.

So while the cost-efficiency of bulk RNA-seq has made tremendous progress by reducing reagent costs per library, relatively little attention has been devoted to quantifying and optimizing how many usable reads, i.e., reads that are used to quantify gene expression levels, are actually produced per library. In general, RNA-seq libraries can differ enormously in that respect, as e.g., seen by an analysis of *>*2,000 tumor bulk RNA-seq datasets across *>*40 studies in which the fraction of usable reads ranged from 0% to 79% at a median of 50%.^25^ Early barcoding protocols show similar patterns with substantial variation among protocols and among samples. For instance, increasing the number of usable reads was a main motivation to develop BRB-seq from the previous SCRB-seq protocol,^11^ and when using prime-seq (another SCRB-seq derivative), we also observed substantial variations in the number of usable reads among samples and especially among sample types. In particular, we observed that some sequencing runs on patterned flow cells of illumina’s NextSeq generated fewer usable reads and especially fewer total reads than expected. We had not observed this before on non-patterned flow cells such as illumina’s Hi-Seq, probably because on those flow cells, one can ignore clustering molecules that do not generate sequencing reads when overloading flow cells.

**Figure 1:**
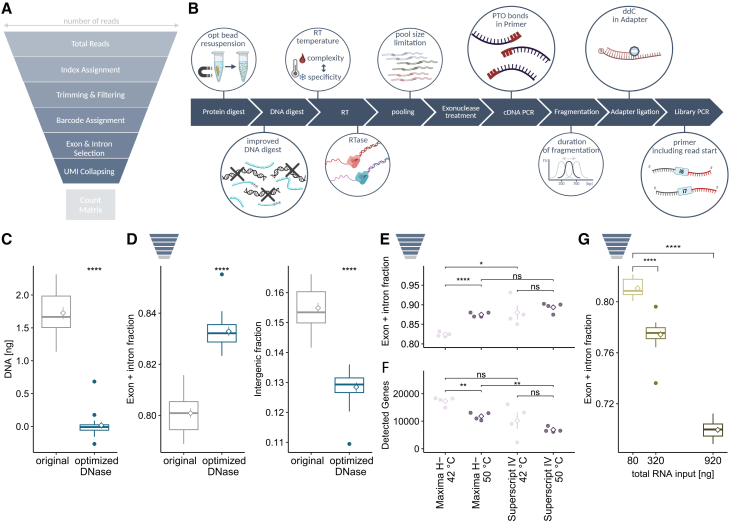
The funnel strategy and its application to prime-seq (A) Funnel strategy scheme. The total reads from the sequencer—having per definition at least a Read1 sequence—are filtered for the presence of both Illumina indices, for the presence of long high quality reads, for the presence of a barcode introduced with the RT-primers, for reads mapping to the coding strand of annotated exons and introns and for reads that differ in their UMI sequence and hence are not PCR duplicates. These UMI counts per gene and per sample constitute the count matrix that is used for all subsequent analysis and hence is the number of usable reads. (B) Overview of the prime-seq workflow and the parameters that have been tested in this study. For a more detailed graphical version, see Figure S7. (C) DNA amount measured after the original DNase treatment under high salt conditions on beads and under the optimized conditions in solution (*n* = 32; two independent replicates of 16 samples each per condition). (D) The fraction of reads mapping to exons and introns (left) and intergenic regions (right) indicates that the optimized DNA digest removes remaining genomic DNA (*n* = 16; 16 samples per condition in one library.). (E) The fraction of reads mapping to exons and introns is lower in the original RT condition (Maxima H- at 42 °C) than in the other tested conditions (*n* = 4 per condition). (F) The number of detected genes, downsampled to 7 million reads per sample, is higher in the original RT condition (Maxima H- at 42 °C) than in the other tested conditions (*n* = 4 per condition). (G) The fraction of reads mapping to exons and introns decreases strongly with increasing amounts of samples pooled after reverse transcription. Samples of 10 ng of total RNA each were reverse transcribed, pooled, exonuclease digested, and then added to the cDNA amplification PCR in amounts corresponding to 8 (80 ng), 32 (320 ng), and 92 (920 ng) samples (total RNA), respectively. This was done in a total of 40 replicates (*n* = 8 for 80 ng and *n* = 16 for 320 ng and 920 ng) from which one library per condition was generated. Boxplots show the median and interquartile range (whiskers: 1.5×IQR). Diamond and whiskers show mean ± SEM. *p* values were calculated in unpaired two-sided t-tests (ns: *p >* 0.05; ∗: *p* ≤ 0.05; ∗∗: *p* ≤ 0.01; ∗∗∗: *p* ≤ 0.001; ∗∗∗∗: *p* ≤ 0.0001). See also Figures S1–S3, S7, S9, S10, S13, S14 and S15, and Table S4.

To optimize the number of usable reads for prime-seq, we systematically varied different steps in the protocol. In this way, we could increase the number of usable reads by 60%, importantly, without reducing gene-level library complexity or increasing technical noise of the prime-seq libraries. This new version—prime-seq2—is a considerable improvement in cost-efficiency, reducing sequencing costs per sample, e.g., from $53.1 to $33.2, when generating 10 million usable unique molecular identifiers (UMIs) at $1.50 per million reads, at an unchanged cost of ∼$3.5 per library including RNA isolation. As we think that many commercial or home-brew early barcoding protocols could profit from such an optimization, we present our approach as a generally applicable optimization strategy that visualizes the amount of usable reads at different processing steps as a funnel (Figure 1A).

## Results

### A funnel strategy to systematically optimize the number of usable reads for bulk RNA-seq protocols

When sequencing RNA-seq libraries, a certain number of reads per sample is targeted, often between 5 and 20 million reads, depending on the number and types of replicates and the intended analyses. After pooling and quantification, one loads the required amount of library molecules on the flow cells of short-read sequencers such as illumina’s NextSeq or NovaSeq. This sets the price tag, but how many reads one gets from the flow cell and how many of those can be used for quantifying expression levels depends largely on the molecules in the library and, hence, largely on the RNA-seq protocol.

For example, reads that cluster on the flow cell but lack a functional read primer binding site result in fewer reads than expected. Similarly, molecules lacking indices or barcodes, having inserts not derived from mRNAs or PCR duplicates (identified by having the same UMI), will be filtered out and reduce the number of usable reads that go into the count matrix, i.e., the table in which reads are counted per sample (columns) and gene (rows). Note that we use reads mapping to exons and introns of genes, as it has been shown that intronic reads are generally derived from pre-mRNA and help to quantify gene expression levels.^16^^,^^26^^,^^27^ Our goal was to increase the cost-efficiency of our prime-seq protocol by increasing the number of usable reads without compromising gene-level library complexity (see Table 1 for definitions). To visualize bottlenecks, we found it helpful to use a “read funnel” displaying the number of usable reads retained after each sequential filtering step, a strategy allowing a better visualization and systematic optimization of RNA-seq protocols in general (Figure 1A).

**Table 1:** Definitions of read classes and performance metrics

Read class/metric	Definition (used throughout)
Total reads	all reads returned by the sequencer.
Index-assigned reads	total reads with correct i5/i7 indices.
Trimmed and filtered reads	index-assigned reads after polyA/adapter trimming and length filtering.
Barcode-assigned reads	trimmed and filtered reads with correct barcodes.
Mapped reads	barcode-assigned reads that align to the reference genome.
Intergenic reads	mapped reads mapping to the genome but not to sense-strand annotated exons/introns; tracked as quality control (QC).
Antisense reads	mapped reads overlapping annotated exons/introns but mapping to the opposite strand; part of intergenic reads; tracked as QC.
Exonic/intronic reads	mapped reads overlapping an annotated exon or intron on the annotated (sense) strand.
Usable reads	mapped reads overlapping an annotated exon or intron on the annotated (sense) strand with unique UMI; =UMI collapsed reads; used for quantification.
Gene-level library complexity/detected genes	number of genes with *>*0 usable reads after downsampling each sample to the same sequencing depth.

### Identifying potential optimizations for prime-seq

We had previously developed and benchmarked prime-seq and could show that its power is comparable to TruSeq, making it 4-fold more cost-efficient due to almost 50-fold cheaper library costs.^16^ It uses the principles of poly(A) priming, template switching, early barcoding, and UMIs to generate 3′ tagged RNA-seq libraries (Figures 1B and S7) and hence is similar to 3′ early barcoding bulk RNA-seq protocols such as BRB-seq^11^ or DRUG-seq^20^ and to the 3′ early barcoding scRNA-seq protocol of 10× Genomics.

Prime-seq has been used extensively in our lab over the past years. During that time, and especially with the switch from non-patterned flow cells of illumina’s HiSeq to the patterned flow cells of the NextSeq, we noticed that, for some samples, sequencing yields were 20–30% lower than expected based on illumina’s nominal flow cell output specifications. We also noticed that we often lost a considerable (10–30%) amount of reads for which we couldn’t assign the i7 index, and that some sample types had a very high proportion of intergenic reads (*>*30%). These patterns were not immediately apparent, as they had always been present to some degree and varied substantially across samples. Furthermore, it is not obvious how these patterns are caused and how they could be fixed, given the many interactions among the different protocol steps and sample types.

As even small improvements could provide considerable benefits for a highly used protocol and as testing variations might reveal general mechanistic insights into the generation of sequencing libraries, we tested nine protocol steps that we identified as promising for increasing the number of usable reads (Figure 1B). We iteratively tested these protocol modifications, amounting to a total of 1,256 RNA-seq samples in 65 libraries for a total of 255 million reads that we structured and visualized using our funnel strategy. For initial testing, a mouse whole-brain lysate was used as an abundant, homogeneous sample. Later, lysates from mouse striatal punch biopsies were used as more challenging samples for which we had previously seen a very low fraction of usable reads with the old protocol. In the following, we discuss the tested modifications in the order they appear within the protocol.

### Additional RNA washing increases exonic reads but reduces detected genes

RNA extraction from lysates comprises the digestion of proteins using proteinase K, followed by a DNase treatment. After each step, samples are purified using solid-phase reversible immobilization (SPRI) beads to remove substances such as salts, small nucleic acids, proteins, lipids, or polysaccharides that can negatively impact subsequent steps in the protocol. This is typically done by adding ethanol for a few seconds to the immobilized beads on the magnet. We reasoned that a resuspension of the beads in ethanol at the washing steps might enhance the purification process by removing more contaminants and, in this way, increase the number of usable reads. This additional resuspension increased the mean proportion of exonic reads from 68% to 74% while reducing intronic, intergenic and unmapped reads (Figure S1A). However, additional resuspension also led to a reduction in the mean number of UMIs per read by 2% (Figure S1B). We also observed a non-significant 7% decrease in the gene-level library complexity, i.e., in the number of detected genes at 1.25 million reads per sample, with a larger spread of detected genes among samples (Figure S1C).

Hence, the additional RNA washing increased exonic reads, but led to fewer usable reads in the end and to libraries with fewer detected genes and potentially more variable libraries. We do not know the reasons for these changes, but as the additional washing does not improve the number of usable reads and as it increases hands-on time, we decided not to incorporate this additional washing step into an improved prime-seq protocol. However, it might remain an option worth testing for lysates with more challenging compositions than whole brain lysates, such as samples with a very high lipid content or chlorophyll-containing samples.

### Improved DNase treatment reduces intergenic reads

Funnels from previous prime-seq projects indicated that a considerable fraction of reads is lost at exon and intron assignment due to 10–20% of reads mapping to intergenic regions within the genome, i.e., all reads not mapping to the expected strand of the current version of GENCODE annotated exons and introns.^27^ Reverse transcriptases used for cDNA generation, including the engineered MMLV-type RTs such as Maxima and SuperScript, can generally also use single-stranded DNA as a template^28^ and hence these reads can in principle be derived from contaminating genomic DNA. However, our previous experiments with mixing human total RNA and mouse genomic DNA had shown that the fraction of UMIs derived from mouse genomic DNA is below 5%, even if more than half of the input is mouse DNA and no DNase I treatment is done.^16^ Furthermore, the fraction of UMIs derived from mouse genomic DNA is ∼ 0%, even if more than half of the input is mouse DNA and a DNA digest is done, although this digest is done under suboptimal high salt conditions on beads.^16^ These findings are difficult to reconcile with a high fraction of intergenic reads derived from genomic DNA.

One potential explanation could be the different behavior of an intact, high molecular weight DNA present in the mouse-human test samples and a more degraded, low molecular weight DNA in problematic samples that show a high number of intergenic reads. Larger DNA fragments would be expected to stick more firmly to the beads and, consequently, could be less accessible for RT than smaller fragments. Consequently, samples with fragmented genomic DNA in combination with a partial DNA digest due to suboptimal conditions on the beads could lead to more accessible DNA and hence to more intergenic reads. In contrast, under optimal conditions, DNase I should be able to digest the entire genomic DNA to di-, tri-, and short oligonucleotides,^29^ which are then removed during bead clean-up.

Hence, we tested whether an optimized DNA digest would lead to less DNA and to fewer intergenic reads. For this, we compared conditions without DNase I treatment, with the original DNase I treatment under suboptimal (high-salt) conditions on beads, and with the optimized DNase I treatment under low-salt conditions off beads. Indeed, when performing the prime-seq RNA isolation protocol on the mouse whole brain samples, the DNA content in the purified lysate was reduced from 1.7 ± 0.4 ng DNA (mean ± SD) for the original DNase I treatment to 0.0 ± 0.2 ng DNA for the optimized treatment (Figure 1C). This indicates strongly that the original protocol indeed left residual DNA after DNA digestion, which was eliminated in the optimized digest that lowered RNA content only minimally (Figure S2A). Interestingly, samples without any DNase I treatment contained 0.3 ± 0.2 ng of DNA, i.e., considerably less than for the original DNase I treatment. This supports that high molecular weight DNA indeed binds strongly to beads and hence cannot be eluted very well.

To assess the impact of the improved DNase conditions on the final data, we produced libraries from the mouse brain lysates with and without this optimization. As hypothesized, this reduced the fraction of intergenic reads compared to the original protocol from 16% to 13% and accordingly increased the fraction of exonic and intronic reads from 80% to 83% (Figure 1D). Assuming that the optimized DNA digest is complete, as supported by the absence of DNA after elution and as expected from the DNase I activity under these conditions, the remaining intergenic reads must be derived from reverse transcribed RNA (see discussion later in discussion).

In summary, we were able to optimize the genomic DNA digestion within the protocol without additional costs. For our mouse brain samples, this increased the number of usable reads by 3%, but we expect that the improvement can also be considerably bigger for samples that contain more DNA and more partially degraded DNA. Hence, we integrated this optimized DNA digest into our protocol.

### Reverse transcription as a trade-off between priming specificity and gene detection

Reverse transcription (RT) is a crucial step in RNA-seq workflows, preceding amplification by PCR- or *in vitro* transcription. To prime RT of mRNAs, prime-seq currently uses an oligo(dT)_30_ primer with a VN-anchor and the RTase Maxima H- at 42 °C. However, the manufacturer suggests a reaction temperature of 50 °C for this enzyme when utilizing oligo(dT)_18_ primers. Increasing the temperature during RT is expected to enhance priming specificity and to facilitate the denaturation of RNA secondary structures, thereby improving access to more mRNAs.^30^ Additionally, other RTases could improve specificity and diversity of the generated cDNAs, such as the generally high-performing SuperScript IV.^31^^,^^32^^,^^33^^,^^34^

To assess the influence of RTase and temperature, we compared Maxima H- and SuperScript IV at 42 °C and 50°C using the mouse brain lysate. This resulted in 6% and 4% more exonic and intronic reads when using Maxima H- and SuperScript IV, respectively. Similarly, substituting Maxima H- with SuperScript IV resulted in 4% and 2% more exonic and intronic reads at 42 °C and 50°C, respectively.

In total, switching from Maxima H- at 42 °C to SuperScript IV at 50 °C elevated the mean exonic and intronic read fraction from 82% to 89% (Figure 1E). However, these enhancements also led to a decreased number of detected genes. At a sequencing depth of 7 million reads per sample, increasing the temperature alone reduced the mean number of detected genes by 32%. Switching the enzyme to SuperScript IV further decreased the mean number of detected genes by 42% (Figure 1F). As expected, the genes that were not detected after switching RT conditions are expressed at a lower level and are mostly randomly distributed across biological functions (Figure S9). Pre-fragmentation cDNA showed largely comparable size profiles across conditions on a bioanalyzer with the exception of a modest additional peak at 400–450 bp for both enzymes at 50 °C (Figure S14). cDNA yield was considerably reduced at conditions other than Maxima H- at 42 °C, requiring up to 6 additional PCR cycles before library generation and likely also contributing to the higher UMI duplication rate under these conditions (Figure S10).

In summary, increasing the reaction temperature or switching to SuperScript IV led to a higher fraction of usable reads, but at the expense of cDNA yield and gene-level library complexity. This likely results from increased priming specificity under these conditions, highlighting the inherent trade-off between specificity and gene-level library complexity and underscoring the importance of considering multiple quality metrics when optimizing a protocol. Higher specificity may be advantageous for targeted, gene-specific applications, particularly when assessed by gel electrophoresis rather than sequencing. In contrast, for genome-wide RNA-seq, lower specificity combined with higher cDNA yield and broader gene detection is probably preferable (see also discussion), although defining the optimal balance would require more extensive experimentation. In our experimental setup, the 8% increase in usable reads does not compensate for the substantial loss in detected genes and cDNA yield. We therefore chose to proceed with Maxima H- at 42 °C.

### Pooling fewer samples increases the number of usable reads

The key advantage of early barcoding methods is the pooling of cDNA after reverse transcription for PCR amplification and library construction, which reduces handling time, reagent costs, and technical variation. However, multi-template PCRs are known to be prone to technical artifacts such as chimera formation and biased amplification,^35^ and the type and number of artifacts are expected to depend on the number of PCR cycles, the composition of the template pool, and the amount of template. The prime-seq protocol already minimizes the number of PCR cycles, and the composition of the template pool is given by the analyzed samples and hence also not optimizable. But as we occasionally observed lower fractions of usable reads when pooling more samples, we tested whether the amount of template in the cDNA, i.e., the number of pooled samples, affects the number of usable reads.

To investigate this effect, we isolated RNA and set up RT reactions from 132 mouse brain lysate aliquots that contain ∼ 10 ng of total RNA each. We pooled 8, 32, and 92 of them for the subsequent exonuclease treatment, cDNA amplification, and library generation, following the standard prime-seq protocol for these sample sizes. Note that this includes minimizing the number of PCR cycles, which in this case corresponds to 13, 10, and 7 cycles for the pools of 8, 32, and 92 samples, respectively. Strikingly, the increase in input led to a decrease in the mean proportion of exonic and intronic sequences from 81.0% to 70.1% (Figures 1G, S13B, and S13C). In other words, using more cDNA as input in the cDNA amplification leads to fewer usable reads, despite a lower number of PCR cycles, which is considered to reduce technical artifacts in multi-template PCRs. The magnitude of this effect was surprising, also because we are not aware of any findings that report an effect of the amount of cDNA input on the quality of bulk or single-cell RNA-seq libraries. We hypothesized that the decrease in usable reads in larger pools was due to the increased molecular diversity in the cDNA amplification. Therefore, we investigated whether delaying the pooling until after the cDNA amplification rescues the pooling effect (Figure S13A). Surprisingly, samples with delayed pooling showed a similar reduction in usable reads as pooling right after the RT. This suggests that the negative effects of large pools are actually caused during the library PCR.

Analyzing the effect in more detail, we found that the decrease in usable reads was caused almost entirely by an increase in intergenic reads (Figure S13C), and this increase was also caused by reads mapping to the opposite, i.e., antisense, strand of exons and introns, increasing from 3% in the 80 ng condition to 11% in the 920 ng condition (Figure S3A). In general, antisense reads have been attributed to off-target priming, i.e., to priming of the oligo(dT) primer and the template-switching oligo (TSO) primer to sites other than the polyA tail and the 5′ end of the cDNA, respectively.^36^ Off-target priming is prevalent in essentially all bulk and single-cell/single-nucleus RNA-seq libraries, and a recent analysis found that on average 18% (max. 30%) of UMIs are antisense among the seven analyzed 10× Genomics datasets.^37^ So off-target priming in general and antisense reads in particular are a common source of non-usable reads in early barcoding protocols such as prime-seq.

Treating reads antisense of exons or introns as “usable reads” increases gene detection (Figures S8A and S8B). Remarkably, this inclusion also improves the power to detect differential expression (Figure S8C). We discuss the causes and consequences of off-target priming in more detail later in discussion. Of relevance here is that there is no obvious mechanistic explanation why antisense reads are more prevalent when pooling more samples, although an increased amplification bias for shorter molecules could play a role (Figure S3B). Furthermore, reads from larger pools are more often unaligned (Figure S13F) and intergenic reads from larger pools are more often soft-clipped or mismatch-containing (Figures S13G and S13H), suggesting more reads deviate from the expected structure/sequence in larger pools.

Independent of the mechanism, our findings indicate that pooling fewer samples is better for prime-seq and potentially also for other early-barcoding protocols. Hence, our previous recommendation of pooling up to 384 samples of 4–40 ng RNA each^16^ should not be followed. Rather, we recommend that pooling should be limited to ∼ 48–96 samples with ∼ 500 ng of total RNA as input, as this seems a reasonable ad hoc compromise between the time and cost savings of pooling and the disadvantage of less usable reads.

### Fragmentation time is robust to input amounts

After its amplification, full-length cDNA needs to be fragmented to lengths compatible with short-read sequencing technologies such as illumina. If the fragments are too long, the sequencing quality degrades^38^ and clustering efficiency on the flow cell decreases.^39^ Conversely, if the fragments are too short, the sequencing cycles may exceed the insert length, leading to unwanted sequencing of adapter sequences.^40^ The prime-seq adapter sequences are 197 bp (including 12 bp barcode and 16 bp UMI), and we sequence the cDNA insert with only one read of 93 bp, so we aim for a total fragment size of 290–650 bp (Figure S7). Prime-seq uses enzymatic fragmentation (NEBNext Ultra II FS) and double-sided SPRI bead purification to obtain this range of fragments. It is desirable to retain as much of the molecular diversity present in the amplified full-length cDNA also within the fragmented cDNA, and hence to adapt fragmentation time to maximize DNA yield in the size range of 250–650 bp.

We tested 5, 15, and 80 ng of full-length cDNA for fragmentation times of 2–8 min and determined the DNA yield in the desired size range on a bioanalyzer (Figure S4). We found that the 5 min of fragmentation time used for our original protocol works well for 5 ng and the recommended 15 ng, with a maximum yield of 66% DNA mass at 4.5 min. At an input of 80 ng cDNA, fragmentation for 5 min yielded 60% DNA yield, and a maximum of 67% was obtained when fragmenting for 7 min.

Hence, the originally recommended 5-min fragmentation time for 15 ng cDNA works well and is reasonably robust even when 3-fold higher or lower cDNA amounts are used. Slightly longer fragmentation times are optimal for higher cDNA amounts, but as cDNA concentration is measured before fragmentation, we keep the recommendation of using 15 ng cDNA and 5-min fragmentation also in prime-seq2.

### Improved adapter ligation and library PCR increase the number of usable reads

**Figure 2:**
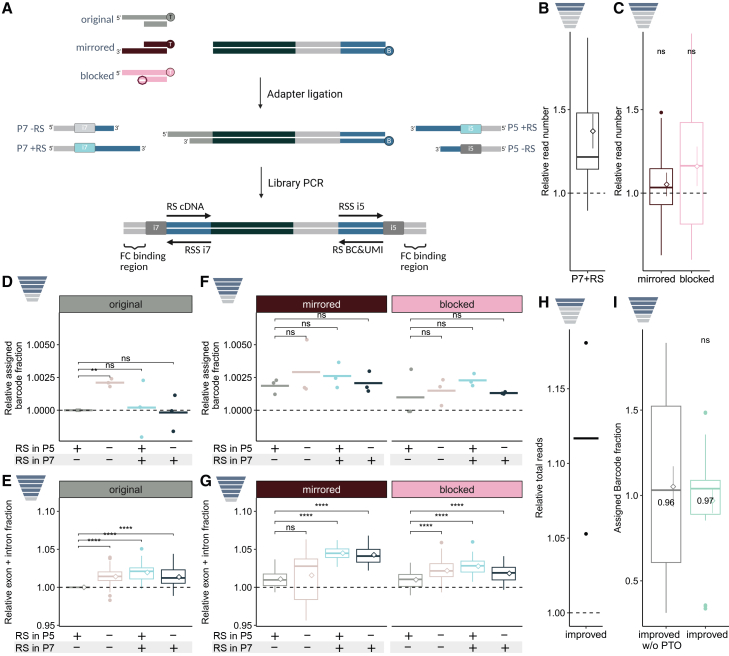
Possible improvements during adapter ligation and library PCR (A) Principle of adapter ligation and library PCR. Adapters are ligated to the cDNA fragments after fragmentation using a 3′ A-overhang. Note that the biotinylated SINGV6 primer prevents ligation on the P5 site (B—Biotin). Alternative mirrored and blocked ligation adapters were designed to prevent the formation of P7-P7 fragments in the following library PCR that amplifies fragments and adds indices (i5 and i7) as well as the complete flow cell adapter sequences (FC binding regions P5 and P7). In the original protocol, a P5 primer with and a P7 primer without a read start (P5+RS and P7-RS) were used, and we tested all four primer combinations with and without a read start for all three adapters with 24 samples per condition and three independent library preparations each, totaling 864 samples. (B) Median ratio of read numbers after index assignment with the new P7+RS relative to the original P7-RS. (*n* = 18 pairs). (C) Median ratio of read numbers after index assignment with each new ligation adapter relative to the original ligation adapter. (*n* = 24 pairs each). (D) Mean fraction of assigned barcodes per combination of index primers with the original ligation adapter (*n* = 3 independent library replicates per condition; bar indicates mean.). (E) Mean fraction of mapped exonic and intronic reads per combination of index primers with the original ligation adapter (*n* = 72 samples per condition). (F) Mean fraction of assigned barcodes per combination of index primers with the new ligation adapters (*n* = 3 independent library replicates per condition; bar indicates mean.). (G) Mean fraction of mapped exonic and intronic reads per combination of index primers with the new ligation adapters (*n* = 72 samples per condition). (H) Total reads relative to the improved protocol without PTO bonds (*n* = 2 independent library replicates per condition. Bar shows the mean; error bars show maximal/minimal values.). (I) Fraction of assigned barcodes with and without PTO bonds. Values are the mean fraction of assigned barcodes per trimmed reads (*n* = 11 (improved)/10 (improved w/o PTO); Two independent library replicates of 10/11 samples per condition.). Boxplots show the median and interquartile range (whiskers: 1.5×IQR). Diamond and whiskers show mean ± SEM. *p*-values were calculated in paired two-sided t-tests (ns: *p >* 0.05, ∗: *p* ≤ 0.05, ∗∗: *p* ≤ 0.01, ∗∗∗: *p* ≤ 0.001, ∗∗∗∗: *p* ≤ 0.0001). See also Figure S5 and Table S4.

To generate an illumina sequencing library from these cDNA fragments, it is necessary to add indices (i5 and i7) and the complete flow cell adapter sequences (P5 and P7) that are necessary for illumina sequencing. As Prime-seq is a 3′-tagged early barcoding method, only cDNA fragments containing the 3′ end can be utilized, as only these contain the oligo(dT) primer with the sample-specific barcode, the UMI, and the partial P5 sequence for priming. To generate an illumina sequencing library from these cDNA fragments, one performs end repair and dA-tailing before ligating an adapter to the 5′ end (Figures 2A and S7). This adapter ligation generates DNA molecules that are amplified in the following “library PCR” that adds the indices (i5 and i7) and the complete flow cell adapter sequences (P5 and P7) necessary for illumina sequencing (Figure 2A). We suspected that the adapters and the indexing primers are suboptimal and contribute to unusable reads in the library. As interactions among adapter ligation and indexing primers are likely, we also tested the combination of these modifications. To minimize batch effects and biases, we started from one homogeneous cDNA pool and constructed three independent libraries per condition with 24 replicates each for all 12 combinations of adapters and primers, totaling 864 samples.

For the indexing primers used in the original prime-seq protocol, we noticed that they might be suboptimal as their annealing sites differ considerably in length, resulting in a melting temperature (Tm) difference of approximately 6 °C. These primers were retained from prime-seq’s predecessor protocol, SCRB-seq,^41^ and while we are not aware of any positive consequences of this Tm difference, it is known that it can lead to inefficient and biased amplification.^42^ To test whether an optimization of the library PCR could improve the number of usable reads, we tested four indexing primer combinations that differ by the presence of a read start site and hence in their length and Tm (Figure 2A): The original protocol combines a long P5 primer that contains the read start site of illumina’s Read 1 sequencing primer (“P5+RS”) and a short P7 primer that does not contain the read start site of illumina’s Read 2 sequencing primer (“P7-RS”). We designed the two additional versions, P5-RS and P7+RS, resulting in the four possible primer combinations. We adjusted the annealing and elongation temperature from 65 °C to 72°C for the P5+RS and P7+RS primer combination, and generated libraries from the same mouse brain cDNA.

We find that libraries generated with P7+RS instead of the original P7-RS have 37% more reads with a correct i5 and i7 index (index assignment step), and libraries generated with P5-RS instead of P5+RS have 43.2% fewer (Figures 2B and S5). Hence, the longer primers generate more sequenceable library molecules, confirming that our previous conditions were suboptimal. Importantly, these additional reads generated by P7+RS and P5+RS library PCRs had the same fraction of reads with assigned barcodes (Figure 2D) and even a 2% higher fraction of reads in exons and introns (Figure 2E), indicating that not only more, but also more usable reads are produced when using P7+RS and P5+RS indexing primers.

The ligation adapter used in the original Prime-Seq protocol has a 15-base 5′-overhang, so that the P7 index primer can anneal only after the P5 index primer has elongated a fragment (Figure 2A). This setup is intended to prevent the amplification of P7-P7 fragments that are generated from internal cDNA fragments that have ligation adapters on both ends. However, this design overlooked that the polymerase can synthesize the complementary overhang of the ligation adapter, enabling the priming of P7 index primers without the prior elongation of the P5 index primer.

To address this issue, we developed two variants of ligation adapters (Figure 2A). The first variant is a mirrored adapter, which has a 3′-overhang instead of a 5′-overhang. This variant leads to complete amplicons only when molecules are initially primed and elongated from the P7 site. Initial priming and elongation from the P5 site, as well as P7-P7 fragments, generate “dead-end” amplicons lacking an overhang on the opposite side for P5 binding. The second variant is a blocked adapter, which includes a dideoxycytidine at its 3′-end. This prevents the synthesis of the complementary overhang and hence should lead to the preferential amplification of fragments with a P5 and a P7 end.

The mirrored and the blocked adapters increased the number of reads after index assignment nonsignificantly by 5% and 16%, respectively (Figure 2C). As for the increased number of reads produced by P7+RS compared to P7-RS, this improvement is likely due to a reduction in irregular fragments, which are quantified but fail to produce valid reads. Importantly, these additional reads also had a slightly higher (∼2%) fraction of reads with barcodes (Figure 2F) and an up to 5% increase in exonic and intronic reads (Figure 2G), indicating that not only more, but also more usable sequences are produced. Notably, the mirrored adapter also leads to less variation in the number of assigned reads than the blocked adapter, but this difference is not significant, and we have no mechanistic explanation for it.

In summary, both new ligation adapters perform better than the original ligation adapter regarding the number of sequenceable reads, fraction of assigned barcodes, and fraction of mapped exons and introns. Both perform best when combined with the P7+RS and P5+RS index primers. As the blocked adapter performed slightly better with respect to the number of sequenceable reads and because it prevents, by design, more mispriming than the mirrored adapter, we integrated the blocked adapter and the P7+RS and P5+RS index primers into the prime-seq2 protocol.

### PTO bonds prevent primer editing

Proofreading polymerases are commonly used in RNA-seq protocols due to their 3′-5′ exonuclease activity, which reduces PCR errors.^43^^,^^44^^,^^45^ However, when mismatches occur between the primer and template, these polymerases can modify primer sequences, leading to nonspecific amplification.^46^^,^^47^ Previous studies have shown that incorporating phosphorothioate (PTO) bonds into the primers’ 3′ ends can block this primer editing.^46^^,^^48^ We incorporated PTO bonds into the last two 3′ bonds of all PCR primers (SINGV6, P5+RS, P7+RS) and validated their effects using the lysates from mouse striatal punch biopsies.

We detected a mean increase in total reads of 11.7% when using primers with PTO bonds (Figure 2H), although this comparison was based on *n* = 2. We therefore interpret this as a trend, which may reflect a decrease in irregular fragments resulting from mispriming, which distort quantification but do not produce valid reads as described above. We also noted a 1.4% higher fraction of mean-assigned barcodes with significantly less variation among samples (Levene’s Test, *p <* 0.05; Figure 2I). Although this experiment is not designed to precisely quantify the effect size of PTO incorporation, it confirmed the expected positive influence on read yield and barcode assignment consistency.

Hence, the incorporation of PTO bonds improved the number of usable reads, and we integrated primers with PTO bonds in the prime-seq2 protocol.

### Validation of the prime-seq2 protocol

Finally, we compared the combination of improvements in the protocol with the original prime-seq protocol using lysates from mouse striatal punch biopsies, generating four libraries of 32 samples per protocol, totaling 256 samples. The improved protocol (prime-seq2) includes the optimized DNA digest, the 3′ blocked ligation adapter, a library PCR with P5+RS and P7+RS primers, and their higher annealing temperature and PTO bonds in SINGV6, P5+RS, and P7+RS primers. Note that for both protocols, 32 samples are pooled, and potential additional improvements from pooling fewer samples (Figure 1G) are not included in this comparison.

**Figure 3:**
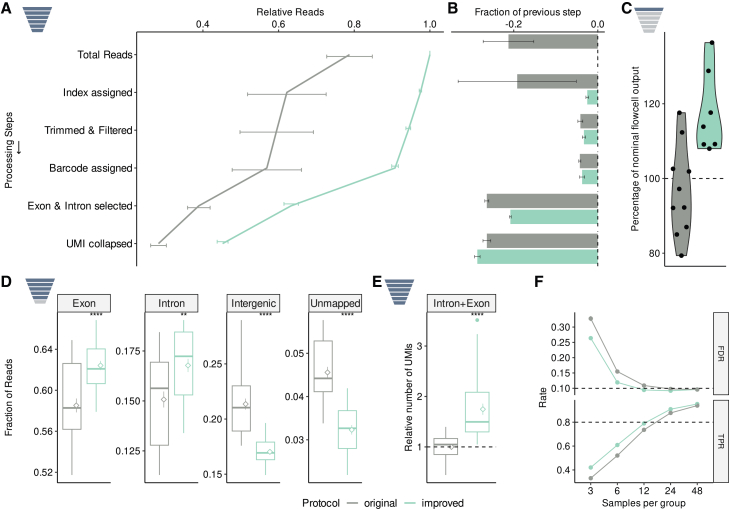
Overall comparison of the improved protocol (A) Funnel plot showing the read number at each step relative to the total reads with the improved protocol. The first four steps use one data point per library, while the last two show one data point per sample, increasing the variation. Lines show means; error bars show SEM. (*n* = 32; Four libraries of 32 samples (eight barcodes) per condition.). (B) Change in total reads per step relative to the previous step. Error bars show SEM. (C) Total reads per flow cell relative to the nominal flow cell output according to the manufacturer. (*n* = 10 (original)/7 (improved)). (D) Fraction of barcode-assigned reads mapped to exons, introns, or intergenic regions and unmapped reads. (*n* = 32; Four libraries of 32 samples (eight barcodes) each per condition.). (E) Number of UMIs relative to the original protocol. (*n* = 32; Four libraries of 32 samples (eight barcodes) each per condition.). (F) Power analysis of both protocols at equal flow cell share across replicate numbers. Points and lines show means; error bars show SEM. Boxplots show the median and interquartile range (whiskers: 1.5×IQR). Diamond and whiskers show mean ± SEM. *p* values were calculated in unpaired two-sided t-tests (ns: *p >* 0.05, ∗: *p* ≤ 0.05, ∗∗: *p* ≤ 0.01, ∗∗∗: *p* ≤ 0.001, ∗∗∗∗: *p* ≤ 0.0001). See also Figures S6, S8, S11 and S12, and Tables S1 and S4.

For both protocols, we visualize the fraction of reads remaining after each step of the read funnel, arbitrarily setting the average number of total reads obtained from the prime-seq2 libraries as 100% (Figure 3A). What matters most is that the number of usable reads at the end of the funnel, i.e., the fraction of UMIs, increases from 28% to 45%, i.e., by 60% (Figures 3A and 3E). Hence, the prime-seq2 protocol represents a considerable improvement in the number of usable reads. Going through each step of the funnel for the total read fraction (Figure 3A) and for the relative change (Figure 3B) helps to explain how this improvement occurs. The new protocol prime-seq2 increases the total reads by a mean of 27%, i.e., for a given amount of library molecules, 27% more reads are obtained from a flow cell. As explained above, we think that this is caused by fewer molecules that get quantified but fail to cluster or—more importantly—cluster, but fail to produce an R1 sequence read and hence do not get detected by the sequencer.

To assess the generalizability of the optimized prime-seq2 protocol beyond mouse brain tissue, we performed additional controlled comparisons using two distinct sample types: mouse liver lysate and human induced pluripotent stem cell (iPSC) lysate. In both cases, libraries prepared using the optimized protocol produced a higher number of initial reads and retained more usable reads throughout the entire downstream processing stage than those prepared using the original protocol (Figure S12), matching the improvements observed in brain and striatal lysates. In addition to these direct comparisons, we observed similar performance gains across 17 unrelated projects from a variety of species and cell types that we sequenced on illumina’s NextSeq1000/2000 using P2, P3, and P4 flow cells (Table S1). The 10 and 7 libraries prepared with the old and the new protocol had 97% and 118% of the expected flow cell output, respectively (Mann-Whitney-U test, *p <* 0.05; Figure 3C). In the next processing step—index assignment 19% of the reads get filtered out in the original protocol, but only 2.5% in prime-seq2. This indicates that more molecules contain a proper i5 and i7 index in prime-seq2. Both protocols then filter a similarly small percentage of reads during trimming and filtering (3–4%) and barcode assignment (4%). This is partly expected, as neither the RT-primer nor the fragmentation and size-selection were changed in prime-seq2, and partly indicates that the PTO bonds do not improve the assigned barcode fraction decisively. The generally small percentage of filtered reads shows that these two steps are not a major bottleneck for the number of usable reads in prime-seq. A considerable fraction of barcode-assigned reads, 26% and 21% for the old and the new version, respectively, is filtered because they do not map to an annotated exon or intron. 4% and 3% cannot be mapped at all, and 21% and 17% map to intergenic regions (Figure 3D). Hence, prime-seq2 not only produce 60% more barcode-assigned reads (Figure 3A), but among them is a significantly higher fraction of exonic and intronic reads. This is probably caused largely by the improved DNA digest (Figure 1D) and the new index primers (Figure 2E). Why, despite a presumably complete DNA digest, the fraction of intergenic reads is still at 17% is interesting and discussed in more detail later in discussion. In the final UMI collapsing processing step, 26% and 29% of the exonic and intronic reads are filtered out. Hence, there is a slightly higher fraction of duplicated reads in the prime-seq2 protocol for unclear reasons. Importantly and as mentioned in the outset, the final number of usable reads, i.e., the total number of exonic and intronic UMIs, increases significantly from 28% to 45%, i.e., by 60% (Figures 3A and 3E).

To quantify how this improvement translates into performance, we used power simulations as implemented in powsimR.^23^ PowsimR estimates the mean-variance distribution of the data, in our case, of the two protocol versions, and then simulates differentially expressed genes between two conditions to obtain a ground truth against which simulations for a certain number of replicates per condition can be compared. We estimated the mean variance distributions using equal flow cell shares and hence took the increased number of total reads of prime-seq2 into account. We simulated that 10% of genes were differentially expressed, with log-fold changes following a gamma distribution, and varied the number of replicates per condition from three to 48. The prime-seq2 protocol consistently had a higher true positive rate (TPR) in detecting differentially expressed genes across all numbers of replicates. prime-seq2 achieves an 80% TPR with only 13 samples per group, whereas the original prime-seq protocol requires 17 samples to reach the same threshold. Importantly, the false discovery rate (FDR) was not compromised and was actually lower than that of the original protocol for low replicate numbers and similar at higher replicate numbers (Figures 3F and S6). Therefore, the protocol improvements increase the power to detect differentially expressed genes without additional sequencing costs.

## Discussion

Bulk RNA-seq is a powerful method for many biological questions, and its cost-efficiency is a crucial factor, limiting biological insights at a given budget. Early barcoding protocols have reduced library costs per sample by 10- to 50-fold by minimizing reagent costs and increasing throughput.^16^ What has been largely neglected in protocol developments is understanding and improving the number of usable reads that are obtained from RNA-seq libraries. Here, we systematically optimized the 3′ tagged early barcoding protocol prime-seq. This results in the improved protocol prime-seq2 that generates 60% more usable reads and presents one of the most widely used and cost-efficient home-brew bulk RNA-seq protocols available. Further, we think our optimization approach reveals insights and approaches for how to optimize other bulk, single-cell, and single-nucleus RNA-seq protocols. Finally, our results provide insights into the types of molecules that get amplified in such protocols and how this affects the interpretation of RNA-seq profiles. We discuss these issues in turn.

### Powerful and cost-efficient RNA-seq with prime-seq2

While using the original prime-seq protocol^16^ in various projects, we noticed that some samples generated fewer usable reads than expected, especially after switching to sequencing on illumina’s patterned flow cells. We identified three main issues: (1) lower-than-expected total sequencing reads per flow cell, (2) loss of reads due to missing index assignments, and (3) a high proportion of intergenic reads. By systematically testing adjustments of different protocol steps as presented above, the resulting prime-seq2 protocol led to 27% more reads per flow cell, 21% more index assignments, and 7.6% more exonic and intronic reads (Figures 3A–3C). While it is not possible to unambiguously attribute each improvement to specific adjustments, we propose the following plausible explanations:

(i) Lower-than-expected total sequencing reads can arise when quantified library molecules fail to amplify on the flow cell, or when amplified molecules lack a functional Read 1 sequencing primer binding site (Figures 2A and S7). We increased total reads in prime-seq2 by 27% (Figures 3A–3C), likely due to the use of blocked ligation adapters, longer P7 primers, and primers with PTO bonds, as these are expected to improve library amplification specificity and indeed increase the number of usable reads (Figure 2).
(ii) Loss of reads due to missing index assignments can arise if amplified and Read 1 sequenced molecules lack a functional Read 2 (i7) or Read 3 sequencing primer binding site (i5). As above, our improvement of 21% (Figures 3A and 3B) is likely caused by our blocked ligation adapters, longer P7 primers, and the incorporation of PTO bonds (Figure 2), as these should increase the specificity of the library PCR and hence produce more sequenceable molecules.
(iii) Intergenic reads (Table 1) were reduced from 21% to 17% in the improved protocol (Figure 3D). Intergenic reads were reduced by optimizing DNA digest conditions, which seems especially relevant if degraded genomic DNA is present (Figures 1C and 1D, and S2). As no genomic DNA is detectable after the optimized digest (Figure 1C), essentially all intergenic reads in the improved protocol must be derived from RNA. The causes and consequences of intergenic reads are discussed in more detail later in discussion. Here, it is worth mentioning that the biggest reduction of over 10% of intergenic reads is achieved when pooling 8 lysates of 10 ng total RNA each instead of 92 (Figure 1G). Note that this effect is masked in our final protocol comparison, as we pooled 32 samples both for the original as well as the prime-seq2 protocol. We currently do not know how this pooling effect is caused, but the increased amount of antisense reads and the reduced representation of long transcripts (Figure S3) indicate that pooling more cDNA could increase the amplification bias toward shorter molecules in the cDNA amplification and/or the library PCR. This likely also plays a role in other early barcoding bulk and scRNA-seq protocols and hence might be a relevant parameter that can improve early barcoding protocols in general. For the current version of prime-seq2, we recommend limiting the libraries to pools of ∼500 ng of total RNA, which is a reasonable compromise for most of our projects with respect to intergenic reads, reagent costs, and hands-on time.

In summary, prime-seq2 delivers on average 60% more usable reads than prime-seq and hence reduces sequencing costs by an average of 38%. For example, this allows for the generation of bulk RNA-seq libraries of 96 samples at $2.9 per sample, including RNA isolation (Table S2) and sequencing data for another $20-$40 per sample (assuming 5–10 million usable reads per sample and $2 per million paired-end reads). To our knowledge, this makes it the most cost-efficient bulk RNA-seq protocol,^16^ although other early-barcoding bulk RNA-seq protocols are in the same range, and proper benchmarking would be required to compare cost-efficiency quantitatively (Figure S11). Prime-seq is well used by us and others,^49^^,^^50^^,^^51^^,^^52^^,^^53^^,^^54^^,^^55^ and due to its low set-up costs, which essentially comprise the 96 custom oligos for ∼$1,450 (Table S2), it is an attractive alternative to commercial kits for core facilities and labs that frequently perform bulk RNA-seq. Because prime-seq2 generates full-length cDNA before short-read library construction, it could in principle also serve as a basis for a full-length RNA-seq protocol compatible with long-read sequencing and isoform-level analyses. However, establishing such an application would require additional protocol development and validation and was therefore outside the scope of the present study. The detailed step-by-step protocol of prime-seq2 is accessible on protocols.io: https://doi.org/10.17504/protocols.io.14egn97kpl5d/v1^56^ and a summary of the changes from the original protocol can be found in Table S3.

### Monitoring and optimizing RNA-seq protocols using the funnel

Our strategy to monitor and optimize prime-seq could also be useful for other RNA-seq protocols, as the number of usable reads varies widely within and among other bulk and single-cell RNA-seq protocols25,57, and principles such as poly(A) priming, early barcoding, and cDNA and library amplification apply to most of them. In addition, prime-seq2 should be easily adaptable to other short-read sequencing platforms such as AVITI (Element Biosciences), by modifying library preparation steps downstream of full-length cDNA generation. In such adaptations, the funnel strategy could be particularly useful to monitor whether platform-specific chemistry or error profiles affect key performance metrics, including gene-level library complexity. We think that just monitoring the different filter steps from total reads to usable reads in more detail, i.e., by applying the funnel strategy, could be a useful quality control (QC) metric that can identify issues for particular samples, protocols, sequencing platforms, and/or platform-specific library preparation workflows. If appropriate, identified bottlenecks could then be addressed by adjusting individual protocol steps. When optimizing such steps, it is important to also track other performance parameters such as gene-level library complexity or technical noise as was e.g., relevant when we tried optimizing RT-conditions or for prime-seq (Figure 1F). It can also be important to consider non-obvious interactions of sample types and protocol steps as we found for the DNA digest that was apparently working well for samples with high molecular weight genomic DNA contamination16, but turned out to be optimizable for samples with degraded genomic DNA contaminations (Figure 1D). Another illustration of the usefulness of the funnel is that for prime-seq2, still about 29% of the reads are lost in the UMI-collapsing step (Figure 3A). This indicates that measures to reduce PCR duplication, such as increasing the input further, optimizing cycle number, and amplification conditions, might be a bottleneck that is worth addressing next.

In summary, we think that the funnel can be a useful QC metric to monitor and optimize bulk and scRNA-seq protocols as well as adapting them to alternative short-read sequencing platforms. It also highlights the intricacies of the conditions and interactions that shape RNA-seq library composition, with implications for how these data should be interpreted.

### Priming, mispriming, and gene detection

Our optimizations also shed light on another aspect of polyA-primed RNA-seq protocols that we think is worth discussing, which is the relation between the specificity of the oligo(dT) priming and the complexity of the resulting libraries. The idealized view is that oligo(dT) priming on the polyA tail of an mRNA leads to RT of the (entire) mRNA and is followed by template switching and second-strand synthesis. The following cDNA amplification and library PCR leads to library molecules in which—for 3′-tagged protocols, the 3′ end of the cDNA is read, starting ∼200 bp downstream of the polyadenylation site (Figures 1B and S7). Due to only one priming site—the polyA tail—the number of reads is independent of the length of the mRNA.

However, stretches of adenine also occur within transcripts (“internal polyA”), which inevitably leads to internal oligo(dT) priming, for which already six or seven consecutive adenines seem sufficient.^57^ This off-target priming is prevalent in bulk and single-cell RNA-seq data and ranges from *<*5% to 65% for exonic reads and *>*95% for intronic reads, dependent on the protocol and the sample (Table S1 in Pförtner et al.^57^). As intronic reads are derived largely from nascent, i.e., (partially) unspliced transcripts, and those are confined to the nucleus, the use of intronic reads is especially relevant for single-nucleus RNA-seq^27^^,^^58^). But also for single-cell data, intronic reads can account for over 30% or over 50% of all reads from mouse embryonic brain samples or human peripheral blood mononuclear cells (PBMCs), respectively.^36^ This creates a dependency between the number of reads and the occurrence of internal poly(A) stretches that in turn leads to the observed gene length bias, i.e., a correlation of number of reads and transcript length.^57^^,^^59^^,^^60^ This reduces the correlation of read counts and the number of RNA molecules in the samples, i.e., reduces the accuracy of the RNA quantification. It has been proposed to filter or correct these off-target reads,^57^ but this does not work well for real data.^60^ However, most bulk and single-cell analyses do not profit from a slightly higher accuracy, as they focus on comparing relative RNA levels among samples or cells to identify differentially expressed genes or distinguish different cell types. For those purposes, library complexity is much more relevant, as it determines how many genes are detected at a given sequencing depth and are therefore available for differential expression analysis. As it turns out that thousands of genes are largely or even exclusively detected by intronic reads,^61^ off-target reads are now considered more as a feature than as a bug, exemplified by intronic reads now being the default for single-cell and single-nucleus data in the 10× CellRanger software.^62^

In the context of this study, this matters, as we not only assumed that intronic reads are usable, but we also observe a relationship of priming specificity and gene-level library complexity: When increasing the temperature for the RT reaction, we do increase the number of usable, i.e., exonic and intronic reads by 6% (Figure 1E), but we also decrease the gene-level library complexity by detecting only 2/3 of the genes (Figure 1F). As alluded to above, this can be explained if the higher temperature reduces off-target priming for many genes that are not well detected by on-target oligo(dT) priming.

Also, other protocols probably differ considerably in off-target priming rates, as seen by the differences in library complexity and fraction of intronic reads among commercial scRNA-seq protocols.^63^ For example, the 5′ expression kit from 10× has three times fewer intronic reads than the 3′ expression kit.^36^^,^^63^ This is likely caused by the lower off-target priming rate of the TSO compared to the oligo(dT)^36^ and this likely leads to the lower number of detected genes per cell (∼2,000 and ∼3,000 in 5′ and 3′ PBMC data, respectively^63^), as a similar reduction in gene number is seen when counting solely exons in 3′ expression data.^62^ While the balance between off- and on-target priming seems important for the performance of RNA-seq protocols, it is currently unclear where an optimal balance might lie, how it could best be measured, and whether it should and could be optimized. In any case, it is likely that it is a major factor contributing to differences in gene expression levels among protocols,^22^ among RNA isolation methods,^16^ or usable reads as discussed later in discussion.

In summary, we find for prime-seq that more stringent RT leads to slightly more usable reads, but fewer detected genes. This stresses the general importance of optimizing not one parameter in isolation and likely reflects the balance between off- and on-target priming, which is an important but largely unexplored parameter for RNA-seq libraries.

### Usable and unusable reads in RNA-seq

Barcode-assigned reads (“reads” in the following) are mapped to the genome and are used further if they map to the sense strand of an annotated exon or intron. For the most recent mouse gene annotation GENCODE34^64^ that is used here, these are 21,414 annotated protein-coding genes, 15,131 long non-coding RNA genes, 6,105 small non-coding RNA genes, and 13,754 pseudogenes that amount to 19.5% of the mouse genome. A considerable fraction of reads—17% for the prime-seq2 validation data (Figure 3D)—map to intergenic regions, and their origin and information content seem worth discussing. Several possible sources for these intergenic reads can be distinguished:

1. Genomic DNA: This is a potential source of intergenic reads as the reverse transcriptase can use DNA as a template. However, as discussed above, this source is probably negligible for prime-seq2 due to its optimized DNA digest.
2. Mismapping: A substantial fraction of reads in RNA-seq— ∼10% of reads for our data—map equally well to 2–50 different regions of the genome (>50 are filtered out). There are different possibilities to handle such multimapped reads,^65^ and STAR randomly assigns them to one region. Among the intergenic reads, 2.9 pp have an equally good match in an exonic or intronic region (data not shown). Assuming that such reads actually do originate from an annotated gene—as currently assumed, e.g., by STARsolo—this would reduce the fraction of intergenic reads to 14.1%.
3. Antisense: For prime-seq2, we find ∼5% of all reads being antisense (Figure S3A), corresponding to ∼9% of all UMIs mapping to exons or introns. In 10× scRNA-seq data, on average 14% of all UMIs mapping to exons or introns are antisense.^36^ Antisense reads may arise from true antisense transcription or from technical artifacts of sense mRNAs. The latter can be caused by self-priming from hairpin structures or by re-priming from RNA fragments.^66^ For 3′-tagged 10× libraries, antisense reads have been attributed to second-strand synthesis initiated by the oligo(dT) primer hybridizing to internal poly(A) stretches on first-strand cDNA.^36^ This generates one sense and one antisense molecule in the library, indeed leading to a correlation of sense and antisense reads that is even higher than the exon-intron correlation.^36^ But as these artifacts are not directly distinguishable from genuine antisense transcription, the current practice is not to use them, as reflected in the default settings of CellRanger and STARsolo. Notably, our simulations indicate that incorporating antisense counts in prime-seq substantially increases statistical power (Figure S8). While requiring further investigation, this indicates that including antisense reads for quantifying sense transcripts, as necessarily done for all unstranded RNA-seq protocols, may represent a promising strategy to increase the fraction of usable reads.
4. Non-annotated readthrough isoforms: It has been shown that thousands of genes that are detected by *in situ* hybridization in a mouse brain region have reads from a 10× experiment of the same region mapping within 10 kb downstream of their annotated 3′ end.^61^ These reads make up 25% of all intergenic reads in this dataset and ∼2,000 genes are only detected when using these reads.^61^ A good proportion of these reads might be derived from short-lived readthrough transcripts that arise especially during stress,^67^ but readthrough transcripts are also found for 1/3 of all protein-coding genes in “normal” human tissues.^68^^,^^69^^,^^70^ Either way, they can be regarded as non-annotated isoforms of the upstream genes and, similar to using introns, increase the number of usable reads and detected genes. Also in our data, we find ∼25% of all intergenic reads to map in sense orientation to regions 10 kb downstream of annotated genes (data not shown), suggesting that a good fraction of intergenic reads might be derived from non-annotated readthrough transcripts. While it has been suggested to incorporate such reads for quantification^61^ and recent ENCODE versions have taken this up and integrated options for extending human and mouse gene annotation,^71^ it is not the current practice to use those reads.
5. Pervasive transcription: After microarrays and next-generation sequencing had made deep transcriptome profiling possible, it became clear that a large fraction of the genome is transcribed to some extent.^72^ This includes the above readthrough transcripts, transcripts originating from enhancers (eRNA) or promotors (PROMPTS), and gives rise to the 36,172 currently annotated long non-coding RNAs^71^ and many more non-annotated ones.^73^ The overly enthusiastic functional interpretations of these transcripts have been rightly criticized^74^ and it is not clear how to interpret lincRNAs in quantitative RNA-seq experiments.

In summary, the 17% intergenic reads consist of 2.3 pp of mismapped reads, ∼5 pp of antisense reads, and 4.3 pp of putative read-throughs. Most of these reads are probably directly related to the canonical transcription of (protein-coding) genes and hence probably improve their detection and quantification, similar to introns. However, in contrast to introns, there is no consensus on when and how often these reads might originate from different processes, such as stress-induced readthrough or genuine antisense transcription that might regulate sense transcription.^75^ It is probably due to this uncertainty that they are not used in current practice. However, given that they do help to detect and quantify genes known to be expressed,^61^ further work is warranted to explore whether this practice might be worth changing.

### Limitations of the study

Although prime-seq2 improves the number of usable reads, it is not possible to unambiguously attribute each gain to a single protocol modification, and our mechanistic explanations therefore remain plausible rather than definitive. Most datasets in this study were sequenced at shallow depth because deeper sequencing would not have been cost-feasible and was not required for our read-funnel and QC metrics.

However, we observed the same qualitative trends in more deeply sequenced validation data (Figure 3C). Our cost estimates depend on local reagent and sequencing costs, and we did not perform a comprehensive head-to-head benchmarking across early-barcoding bulk RNA-seq protocols. More generally, optimizing for usable reads can trade off with other performance metrics. For example, increased priming specificity can increase exonic/intronic read fractions but reduce gene-level library complexity, implying that optimal settings may depend on sample type and study goals. While we expect the funnel strategy to be broadly useful as a QC and optimization readout, we did not systematically validate it across a wide range of protocols and sequencing platforms. Finally, prime-seq2 is a 3′ tag RNA-seq method, which provides high efficiency for gene-level quantification but does not capture full-length transcript information and is therefore not suited for applications that require isoform-resolved analyses.

## Resource availability

### Lead contact

Further information and requests for resources and reagents should be directed to and will be fulfilled by the lead contact, Daniel Richter (richter@bio.lmu.de).

### Materials availability

This study did not generate new unique reagents.

### Data and code availability

• The raw data discussed in this publication have been deposited in NCBI’s Gene Expression Omnibus^76^ and are accessible through GEO Series accession number GSE286365 (ncbi.nlm.nih.gov/geo/query/acc.cgi?acc=GSE286365). Processed data files for data analysis and visualization can be accessed at github.com/Hellmann-Lab/prime-seq2 and as a stable version at https://doi.org/10.5281/zenodo.20607835^77^ (key resources table).
• The code for data analysis and visualization can be accessed at github.com/Hellmann-Lab/prime-seq2 and as a stable version at https://doi.org/10.5281/zenodo.20607835^77^ (key resources table).
• Any additional information required to reanalyze the data reported in this paper is available from the lead contact upon request.

## Acknowledgments

We thank Ines Bliesener for lab support and the Enard/Hellmann group for helpful discussions. We thank Dr. Stefan Krebs and the staff of LAFUGA for sequencing services. Some illustrations in the graphical abstract, Figures 1, 2, S7 and S13 were created with BioRender.com. This work was funded by the 10.13039/501100001659Deutsche Forschungsgemeinschaft (DFG, German Research Foundation)—Project Number 238187445—SFB 1123, Subproject Z02 (W E. and E.B.).

## Author contributions

F.P., E.B., W.E., and D.R. conceived the study. F.P., E.B., and D.R. conducted the experiments. F.P. and E.B. analyzed the data. F.P., E.B., W.E., and D.R. wrote the manuscript.

## Declaration of interests

The authors declare no competing interests.

## Declaration of generative AI and AI-assisted technologies in the writing process

During the preparation of this work, the authors used ChatGPT to assist with language polishing and improving the clarity of selected paragraphs. After using this tool, the authors reviewed and edited the content as needed and take full responsibility for the content of the publication.

## STAR★Methods

### Key resources table

**Table:** 

REAGENT or RESOURCE	SOURCE	IDENTIFIER
**Chemicals, peptides, and recombinant proteins**		

Buffer EB	Qiagen	Cat#19086
Buffer RLT Plus	Qiagen	Cat#1053393
Deoxynucleotide Solution Mix	New England Biolabs	Cat#N0447L
DNase I (RNase-free)	Thermo Fisher Scientific	Cat#EN0525
DNase I Reaction Buffer	Thermo Fisher Scientific	Cat#EN0525
EDTA	Sigma-Aldrich	Cat#E7889
Ethanol absolute	Carl Roth	Cat#9065.4
Exonuclease I (E. coli)	New England Biolabs	Cat#M0293S
Thermolabile Exonuclease I	New England Biolabs	Cat#M0568S
IGEPAL CA-630	Sigma-Aldrich	Cat#I8896
KAPA HiFi Hot Start Polymerase	Kapa Biosystems	Cat#07958935001
Maxima H Minus Reverse Transcriptase	Thermo Fisher Scientific	Cat#EP0752
Polyethylene glycol	Sigma-Aldrich	Cat#89510
Proteinase K solution	Ambion	Cat#AM2546
RNase A	Omega BioTek	Cat#AC118
RNase Away	Thermo Fisher Scientific	Cat#7000TS1
Sodium azide	Sigma-Aldrich	Cat#S2002-100G
Sodium chloride (NaCl) 5 M	Sigma-Aldrich	Cat#S5150-1L
*β*-mercaptoethanol	Sigma-Aldrich	Cat#M3148
SuperScript IV Reverse Transcriptase	Thermo Fisher Scientific	Cat#18090010
TE-Buffer (20X, RNase-free)	Thermo Fisher Scientific	Cat#T11493
Tris-EDTA Buffer	Sigma-Aldrich	Cat#93283
Trizma hydrochloride solution	Sigma-Aldrich	Cat#T2694
UltraPure DNase/RNase-free distilled water	Thermo Fisher Scientific	Cat#10977049

**Critical commercial assays**		

High Sensitivity DNA Kit	Agilent Technologies	Cat#5067-4626
QuantiFluor dsDNA System	Promega	Cat#2670
QuantiFluor RNA System	Promega	Cat#E3310
NEBNext Ultra II FS DNA Library Prep Kit for Illumina	New England Biolabs	Cat#E7805S
NEBNext Ultra II Q5 Master Mix	New England Biolabs	Cat#M0544
SPRIselect	Beckman Coulter	Cat#B23317
Sera-Mag Speed Beads	GE Healthcare	Cat#65152105050250

**Deposited data**		

RNA-seq data	NCBI Gene Expression Omnibus (GEO) (Edgar et al.^76^)	GEO: GSE286365; https://www.ncbi.nlm.nih.gov/geo/query/acc.cgi?acc=GSE286365
Code (analysis and visualization)	GitHub	https://github.com/Hellmann-Lab/prime-seq2
Code (archived release)	Zenodo	https://doi.org/10.5281/zenodo.20607835; https://doi.org/10.5281/zenodo.20607835 (Pförtner 2026)

**Experimental models: Cell lines**		

Human iPSC line: KOLF2.1J (HPSI0114i-kolf_2 derived; male donor)	The Jackson Laboratory	RRID:CVCL_B5P3

**Experimental models: Organisms/strains**		

Mus musculus: Foxp2hum/hum (5H11 line; male; 250 days)	Enard et al.^78^	N/A
Mus musculus: C57BL/6J (adult; mixed sex; mean age 5.5 months)	The Jackson Laboratory	RRID:IMSR_JAX:000664

**Oligonucleotides**		

E3V7NEXT	Sigma-Aldrich	N/A
Ligation adapter antisense (5′ phosphorylated)	IDT	N/A
Ligation adapter sense	IDT	N/A
Ligation adapter blocked antisense (5′ phosphorylated; 3′ ddC)	IDT	N/A
Ligation adapter blocked sense	IDT	N/A
P5+RS PTO	IDT	N/A
P7+RS PTO	IDT	N/A
SINGV6 PTO	Sigma-Aldrich	N/A
TSO	Sigma-Aldrich	N/A

**Software and algorithms**		

STAR (v2.6.1c)	Dobin et al.^79^	https://github.com/alexdobin/STAR
SAMtools (v1.11)	Li et al.^80^	http://www.htslib.org/
zUMIs (v2.9.7)	Parekh et al.^27^	https://github.com/sdparekh/zUMIs
deML (v1.1.1)	Renaud et al.^81^	https://github.com/grenaud/deML
cutadapt (v3.2)	Martin^82^	https://github.com/marcelm/cutadapt
BEDTools (v2.30.0)	Quinlan and Hall^83^	https://github.com/arq5x/bedtools2
BEDOPS (v2.4.35)	Neph et al.^84^	https://bedops.readthedocs.io/
R (v4.4.1)	R Core Team^85^	https://www.r-project.org/
tidyverse (v2.0.0)	Wickham et al.^86^	https://CRAN.R-project.org/package=tidyverse
data.table (v1.17.4)	Dowle and Srinivasan^87^	https://CRAN.R-project.org/package=data.table
ggbeeswarm (v0.7.2)	Clarke et al.^88^	https://CRAN.R-project.org/package=ggbeeswarm
purrr (v1.0.4)	Wickham and Henry^89^	https://CRAN.R-project.org/package=purrr
ggpubr (v0.6.0)	Kassambara^90^	https://CRAN.R-project.org/package=ggpubr
patchwork (v1.3.2)	Pedersen^91^	https://CRAN.R-project.org/package=patchwork
cowplot (v1.1.3)	Wilke^92^	https://CRAN.R-project.org/package=cowplot
ShortRead (v1.62.0)	Morgan et al.^93^	https://bioconductor.org/packages/ShortRead/
bioanalyzeR (v0.10.1)	Foley et al.^94^	https://bioconductor.org/packages/bioanalyzeR/
here (v1.0.1)	Müller^95^	https://CRAN.R-project.org/package=here
readxl (v1.4.3)	Wickham and Bryan^96^	https://CRAN.R-project.org/package=readxl
rstatix (v0.7.2)	Kassambara^97^	https://CRAN.R-project.org/package=rstatix
car (v3.1-2)	Fox and Weisberg^98^	https://CRAN.R-project.org/package=car
powsimR (v1.2.5)	Vieth et al.^23^	https://CRAN.R-project.org/package=powsimR
Rsamtools (v2.20.0)	Morgan et al.^99^	https://bioconductor.org/packages/Rsamtools/
GenomicAlignments (v1.40.0)	Lawrence and Pagès^100^	https://bioconductor.org/packages/GenomicAlignments/
GenomicFeatures (v1.56.0)	Lawrence and Pagès^101^	https://bioconductor.org/packages/GenomicFeatures/
GenomeInfoDb (v1.40.1)	Arora et al.^102^	https://bioconductor.org/packages/GenomeInfoDb/
GenomicRanges (v1.56.1)	Lawrence and Pagès^103^	https://bioconductor.org/packages/GenomicRanges/
BSgenome.Mmusculus.UCSC.mm39 (v1.4.3)	Bioconductor Core Team^104^	https://bioconductor.org/packages/BSgenome.Mmusculus.UCSC.mm39/
AnnotationDbi (v1.66.0)	Pagès et al.^105^	https://bioconductor.org/packages/AnnotationDbi/
org.Mm.eg.db (v3.19.1)	Carlson^106^	https://bioconductor.org/packages/org.Mm.eg.db/
rtracklayer (v1.64.0)	Lawrence et al.^107^	https://bioconductor.org/packages/rtracklayer/
clusterProfiler (v4.12.2)	Wu et al.^108^	https://bioconductor.org/packages/clusterProfiler/
UpSetR (v1.4.0)	Conway et al.^109^	https://CRAN.R-project.org/package=UpSetR
Matrix (v1.7.0)	Bates et al.^110^	https://CRAN.R-project.org/package=Matrix
optparse (v1.7.5)	Davis^111^	https://CRAN.R-project.org/package=optparse
ggsci (v3.2.0)	Xiao^112^	https://CRAN.R-project.org/package=ggsci
plotly (v4.10.4)	Sievert^113^	https://CRAN.R-project.org/package=plotly
reshape2 (v1.4.4)	Wickham^114^	https://CRAN.R-project.org/package=reshape2
qvalue (v2.36.0)	Storey et al.^115^	https://bioconductor.org/packages/qvalue/
IHW (v1.32.0)	Ignatiadis et al.^116^	https://bioconductor.org/packages/IHW/
IRanges (v2.38.1)	Lawrence et al.^117^	https://bioconductor.org/packages/IRanges/
limma (v3.60.6)	Ritchie et al.^118^	https://bioconductor.org/packages/limma/
edgeR (v4.2.2)	Robinson et al.^119^	https://bioconductor.org/packages/edgeR/

### Experimental model and study participant details

#### Mouse lines

All procedures involving animals were conducted in accordance with the German Animal Welfare Act (Tierschutzgesetz). Animals were housed and handled at the Biology Faculty Animal Facility at Ludwig Maximilian University in accordance with institutional ethical standards and licensed by the competent authority (Landratsamt München, AZ 4.3.2-5682/LMU). This includes tissue collection without additional experimentation in compliance with § 4 Abs. 3 Tierschutzgesetz as was done for this study. Brain and liver tissue from a 250-day-old male *Foxp2*^*hum/hum*^ mouse (5H11 line^78^) was used. Additionally, striatal punches of adult C57BL/6J mice were used (mixed sex, mean age 5.5 months). As this study focused on methodological optimization, sex was not considered as an experimental variable in the design or analysis, and no sex-specific analyses were performed.

#### Human lines

KOLF2.1J (HPSI0114i-kolf_2, male donor, 55–59 years at sampling) human iPSCs were obtained from a commercial supplier. No additional cell line authentication was performed in this study. The cells did not show any abnormalities during culture, but have not been tested for mycoplasma contamination before lysis. The line was originally derived from donor material collected with informed consent under appropriate ethical approvals.^120^ No new human participants or samples were recruited or collected for this study and no identifiable human data were used.

### Method details

#### Sample preparation

To compare different RNA-seq protocols using the same source material, an abundant, representative tissue or cell lysate was needed. Brain and liver tissue from a 250-day-old male *Foxp2*^*hum/hum*^ mouse (5H11 line^78^) was used. The tissue was homogenized in RLT Plus with 1% *β* -mercaptoethanol and stored at -70°C. For validation, additional, more challenging samples were used, which delivered worse results with the original protocol. Striatal punches of adult C57BL/6J mice (mixed sex, mean age 5.5 months) and KOLF2.1J cells were lysed in RLT Plus with 1% *β* -mercaptoethanol and stored at -70°C. Lysates from multiple mice were pooled to create a homogeneous sample.

Total RNA concentrations were determined using the QuantiFluor RNA System after Proteinase K treatment, DNA digest and purifications using SPRI beads.

#### Prime-seq workflow

The complete step-by-step prime-seq2 protocol including reagents and oligonucleotides can also be found under https://doi.org/10.17504/protocols.io.14egn97kpl5d/v1.^56^ The full list of reagents and oligonucleotides can be found in the key resources table and Table S5.

The RNA-seq workflow used as a starting point is prime-seq.^16^ It is termed as the “original” prime-seq method and used in this way if not mentioned otherwise. It is described in the following section and visualized in Figure S7.

#### Preparation of SPRI beads

Homemade solid-phase reversible immobilization (SPRI) beads are used throughout the paper. They are produced as follows: the 22% PEG solution (22% (w/v) PEG 8000, 1 M NaCl, 10 mM Tris-HCl pH 8, 1 mM EDTA. 0.01% IGEPAL, 0.05% sodium azide, to 49 ml with UltraPure (UP) H_2_O) was heated at 40 °C to dissolve PEG. Sera-Mag Speed Beads (1 ml) were separated from their solution using magnets. The supernatant was removed. The resulting pellet was washed twice in TE buffer (10 mM Tris-HCl, pH 8, 1 mM EDTA) and eluted in 0.9 ml TE buffer. This bead suspension was mixed with the 22% PEG solution. SPRI beads were stored at 4 °C and equilibrated to room temperature before usage.

#### Nucleic acid purification using SPRI beads

Home-made SPRI beads were used for nucleic acid purification at multiple steps during the RNA-seq protocol. In the ensuing procedures, “cleanup” always followed this protocol: Bead suspension was added in the specified ratio, incubated for 5 min, and the beads were anchored using magnets. The supernatant was discarded, and the beads washed twice with 50-1000 *μ*l 80 % ethanol in UP H_2_O. Beads were air-dried, removed from the magnet and eluted in UP H_2_O. After 5 min incubation, beads were anchored again using magnets and the supernatant was transferred to a new well.

#### cDNA preparation

Once the RNA concentration of the standard lysate sample had been established, the standard input of the samples was adjusted to target approximately 10 ng of total RNA per sample. First, proteins were digested using 20 *μ*g Proteinase K in 0.5 mM EDTA at 50 °C for 15 min, followed by heat inactivation at 75 °C for 10 min. SPRI beads were used in a 2× ratio (given as bead volume to sample volume) to purify the samples as described above.

In this step, samples were resuspended in 5 *μ*l UP H_2_O without removing the beads. DNA was digested on beads using 1 unit of DNase I at 20 °C for 10 min in 1× DNase I buffer and 1× bead binding buffer (11 % (w/v) PEG 8000, 0.5 M NaCl, 5 mM Tris-HCl pH 8, 0.005 % IGEPAL, 0.025 % sodium azide). EDTA was added to 4.5 mM before heat-inactivation at 65 °C for 5 min. The samples were cleaned up again and eluted in 4 *μ*l UP H_2_O. RT was performed in 1× Maxima H Minus buffer using 30 units Maxima H Minus, 1 mM each dNTP, 1 *μ*M template-switching oligo (TSO), and 1 *μ*M barcoded oligo (dT) primers (E3V7NEXT). The RT reaction was incubated at 42 °C for 90 min. The samples were combined, purified using SPRI beads at a 1× ratio and eluted in 17 *μ*l in UP H_2_O. This “pooled” sample was termed the “preAmp pool”. Residual primers were digested using 20 units Exonuclease I in 2 *μ*l 10× Exonuclease I buffer at 37 °C for 20 min with ensuing heat inactivation at 80 °C for 10 min. The samples were cleaned using SPRI beads at a 0.8× ratio, and eluted in 20 *μ*l of UP H_2_O. cDNA was amplified in 1× KAPA HiFi Ready Mix and 0.6 *μ*M SINGV6 primer, according to this PCR protocol: initial denaturation at 98 °C for 3 min, denaturation at 98 °C for 15 s, annealing at 65 °C for 30 s, elongation at 68 °C for 4 min, and final elongation at 72 °C for 10 min. Denaturation, annealing, and elongation were repeated for 10–23 cycles, depending on the initial amount of total RNA. The DNA was cleaned with SPRI beads in a 0.8× ratio and eluted with 10 *μ*l of UP H_2_O. The cDNA concentration was measured using the QuantiFluor dsDNA System and the quality of the cDNA was assessed using a Bioanalyzer.

#### Library preparation

Libraries were prepared using the NEBNext Ultra II FS Library Preparation Kit. 1.2-4 μl cDNA (usually 4–20 ng) were fragmented using the FS Enzyme Mix and FS Reaction Buffer in a 6 μl reaction by incubating at 37 °C for 5 min, then at 65 °C for 30 min. 0.5 μl ligation adapter (1.5 μM) was ligated using the Ligation Master Mix and Ligation Enhancer in a reaction volume of 12.7 μl by incubating at 20 °C for 15 min. Double-sided size selection was performed using SPRIselect beads according to the manufacturer, with a bead ratio of 0.52× for the removal of long DNA fragments and a ratio of 0.72× for the removal of short fragments. Samples were eluted in 11 μl 0.1× TE buffer. 10.5 μl DNA sample was amplified using 12.5 μl Q5 Master Mix, 1 μL Nextera P7 index primer (5 μM), and 1 μL TruSeq P5 index primer (5 μM) following this PCR protocol: initial denaturation at 98 °C for 30 s, denaturation at 98 °C for 10 s, annealing and elongation at 65 °C for 1 min 15 s, and final elongation at 65 °C for 5 min. Denaturation and annealing/elongation were repeated for 10–13 cycles, depending on the initial amount of cDNA. Double-sided size selection was performed as above. The initial DNA concentration was measured using the QuantiFluor dsDNA System and, more precisely, using a Bioanalyzer, which also allowed for quality assessment.

#### Prime-seq interventions

The following experiments are all modifications of the original prime-seq protocol described above. Of note, insert size distributions were highly comparable between libraries, with mean fragment sizes of 429 ± 16.7 bp for the original libraries and 422.8 ± 13.9 bp for the modified libraries (mean ± SD; *n* = 5 each). The relative width of the size distributions was also similar (CV = 21.6% vs. 21.7%), consistent with identical double-sided bead clean-up cutoffs.

#### Cleanup resuspension

The cleanup steps following Proteinase K and DNase I digest were altered to include resuspension in ethanol. After adding 80% ethanol, the plate was taken from the magnet, beads were resuspended in ethanol by pipetting, beads were reanchored using the magnet and ethanol was removed. This procedure was repeated in the second wash step.

#### DNase I digest

In order to decrease the NaCl concentration during DNase I digest, bead binding buffer was removed from the reaction, and UP H_2_O was added to keep the reaction volume constant. Consequently, 18 *μ*l bead binding buffer was added after heat-inactivation to obtain the same PEG concentration as with the initially added bead binding buffer.

#### Reverse transcription

The incubation temperature during reverse transcription (RT) was increased from 42 °C to 50°C. The number of PCR cycles during cDNA amplification PCR was increased by six cycles at 50 °C to obtain similar cDNA yields. Additionally, SuperScript IV was tested against the standard Maxima H Minus as an alternative reverse transcriptase (RTase). In this reaction setup, Maxima buffer was swapped for SuperScript IV buffer, and DTT was added to 5 mM, while keeping all other reagents and the total volume constant.

#### cDNA amplification pool size

To test the effect of combined RNA input in the cDNA amplification, pools of 8, 32, and 92 samples were compared. 10 ng total RNA brain lysate was used per sample. Although increasing pool size required proportionally larger absolute bead volumes during the clean-up step after pooling, bead-to-sample ratios were kept constant, such that size selection and recovery characteristics were unchanged. The second experiment investigated whether delaying pooling until cDNA amplification reduces the pooling effects seen in experiment 1. The 80 ng samples were treated as before. For 920 ng, samples were kept in 4 pools of 230 ng until pooling them in different protocol steps. Samples were pooled either directly as before after RT (920 1 1), after exonuclease digest (920 4 1), or after cDNA amplification (920 4 4). This was done in a total of 56 replicates (*n* = 8 for 80 ng and *n* = 16 for each 920 ng condition) from which one library per condition was generated.

#### Fragmentation

To determine the optimal fragmentation time, cDNA from standard brain lysate was processed in the standard prime-seq library preparation without size selection. 5, 15, and 80 ng cDNA were fragmented for 0, 2, 3.5, 4, 4.5, 5, 6, 7, 8, or 15 min. Fragmentation was compared using a Bioanalyzer. The fraction of DNA in the target size range (250-650 bp) relative to the full profile (100-8000 bp) was computed with bioanalyzeR (region.ratio on concentration).

#### Ligation adapters and index primers

Altered ligation adapters and index primers were compared. The mirrored ligation adapter contains swapped strands to have a 3′ overhang instead of a 5′ overhang. The blocked prime-seq ligation contains an added dideoxycytidine at its 3′ end. The P5+RS and P7-RS primers correspond to the primers used in the original prime-seq protocol. The P5-RS primer was shortened by 8 nt from the 3′ end, which decreases its melting temperature from 75 °C to 71°C. The P7+RS primer was extended by 19 nt at the 3′ end to its maximum length, which increases its melting temperature from 65 °C to 74°C. Only the P5/7+RS primers contain the complete start sites for priming of the read 1 and read 2 sequencing reads, respectively. In combinations of P5+RS and P7+RS, Annealing/Elongation temperature during library PCR was increased from 65 °C to 72°C, as calculated using the NEB Tm calculator.

#### PTO bonds

In the cDNA PCR and the library PCR, SINGV6 and index primers (P5+RS and P7+RS) with two 3′-terminal phosphorothioate (PTO) bonds were used.

#### Sequencing

RNA-seq libraries were sequenced by the Blum Lab as part of the Laboratory for Functional Genome Analysis (LAFUGA) of the Gene Center Munich. Sequencing was performed on an Illumina NextSeq 1000/2000 instrument with the following setup: Read 1: 28 bp, Index 1: 8 bp, Index 2: 8 bp, Read 2: 93 bp. A breakdown of the number of reads obtained after demultiplexing can be found in Table S4.

To estimate the required sequencing depth for a given experimental design, power analysis frameworks such as powsimR^23^ can be used to simulate RNA-seq count distributions and determine the number of reads needed to achieve adequate statistical power for detecting differential expression under defined assumptions. However, since key parameters such as expected effect sizes are often unknown *a priori*, we recommend using a practical starting point of approximately 10–15 million reads per sample for typical differential expression studies with 6 biological replicates per condition and moderate effect sizes.

The fraction of usable reads in unrelated projects (Table S1) is on average lower than in our validation data. This is due to differences in sequencing depth which strongly affects the ratio of reads to UMIs. Thus, the unrelated projects and the validation data are more comparable when using the fraction of intronic and exonic reads before UMI collapsing.

#### DNase digest test

DNase digest was compared using the original protocol, the improved protocol, and a negative control lacking DNase I. 8 samples per condition of mouse brain lysate containing 50 ng total RNA each were used. After Proteinase K treatment, the DNase I digest was done as in the original protocol, without DNase I and using the improved low-salt conditions. RNA was quantified using the QuantiFluor RNA System. RNA was digested using 1 *μ*l RNase A per sample at room temperature for 90 min. DNA was quantified using the QuantiFluor dsDNA system after a bead clean-up. The experiment was independently repeated.

### Quantification and statistical analysis

#### Data analysis

Prime-seq libraries were demultiplexed with deML (v1.1.1^81^) using the indices in the index primers. Terminal poly(A)s were trimmed from read 2 and read pairs were filtered to minimum lengths of 28 nt read 1 and 40 nt read 2 using cutadapt (v3.2^82^): “cutadapt -A A{30} –discard-casava -minimum-length 28:40”. zUMIs (v2.9.7^27^) was used for further processing. Reads were filtered if more than 2 barcode bases had Q-scores below 20 or if more than 3 UMI bases had Q-scores below 20. Barcodes (read 1, 1-12 nt) were demultiplexed and binned within a Hamming distance of 2. Remaining reads were mapped to the genome GRCm39 and GRCh38. Exonic and intronic reads and UMIs (read 1, 13-28 nt) were counted per gene according to mouse Gencode version 34 and human Gencode version 48. Read classes (total reads, index-assigned, trimmed & filtered, mapped, barcode-assigned, exonic/intronic, UMI-collapsed) were aggregated per library or per sample as appropriate to build the read funnel (Table 1); funnel plots show reads retained after each step, with the final UMI count defining usable reads used for quantification.

#### Power simulations

Power simulations were performed with powsimR (v1.2.5^23^). For each protocol, the same flow cell share was used for power simulations. This is not the same number of reads, since the improved protocol produced more reads for a given flow cell share (Figure 3C). For each protocol and sample size setup (3 vs. 3, 6 vs. 6, 12-20 vs. 12-20, 24 vs. 24, and 48 vs. 48), 25 simulations were performed with a negative binomial distribution, trimmed mean of M values (TMM) normalization, limma-voom DE-method, and 10% differentially expressed genes. The log fold change was modeled by a gamma distribution with shape = 1 and rate = 2. Library (batch) was included in the design when estimating the mean–variance relationship. The data were stratified by absolute log fold change or mean expression. To quantify sense+antisense power, the antisense count matrix was added to the sense count matrix and subset to genes already expressed in the count matrix.

#### Downsampling

Where library depth differed between conditions (e.g., pool-size, RT conditions, protocol comparison), counts or BAMs were downsampled to a common depth before comparing complexity, fractions, or power so that differences were not driven by sequencing depth. Seeds were fixed for reproducibility.

#### Library complexity (detected genes)

Gene-level complexity was defined as the number of genes with at least one UMI. For comparisons across conditions, data were downsampled to the same depth per sample, then the number of genes with ≥1 UMIs was computed. To test whether gene-level complexity estimates were driven by genes supported by only a single UMI, we repeated the detected-gene analyses using more stringent thresholds of ≥5 and ≥10 UMIs per gene (Figure S15). Gene-level counts from zUMIs (exon + intron) were used.

#### Antisense and intergenic reads

For pool-size and related analyses, antisense and intergenic read fractions were obtained from zUMIs output (Exon, Intron, Intergenic, Unmapped, Ambiguity). To quantify antisense intergenic reads specifically, libraries were recounted with zUMIs with library strandedness set to reverse. Intergenic read quality (soft-clipping fraction, mismatch rate) was extracted from BAMs using Rsamtools and GenomicAlignments for pool-size comparisons (Figure S13). To quantify intergenic reads in sense orientation within 10 kb downstream of annotated gene ends (putative read-through), BAMs were overlapped with gene ranges extended 10 kb downstream; distances and per-gene counts were summarized (quantify downstream scripts).

#### UMI duplication rate

UMI duplication rate was defined as 1 minus the ratio of unique UMIs to total exonic/intronic reads (i.e. reads lost in the UMI-collapsing step). For the RT temperature comparison (Figure S10), BAMs were downsampled to equal assigned read depth per condition, then per-barcode read and UMI counts were obtained from BAM tags (BC, UB, GE, GI) and the duplication rate was computed per sample.

#### Multimapped reads

STAR assigns reads with multiple equally good alignments to one locus at random. The fraction of mapped reads that had multiple primary (best) alignments, and their annotation as exon, intron, or intergenic, was quantified from STAR BAMs and BED overlap (quantify miscounting scripts) for interpretation of intergenic and multimapper fractions.

#### Gene length and transcript choice

For analyses that required a single representative transcript per gene (e.g. length binning in pool-size experiments), transcripts were chosen in this order: (1) consensus coding sequence (CCDS), (2) highest GENCODE support level, (3) longest transcript.

#### Gene set enrichment

Gene set enrichment was performed with clusterProfiler. For the reverse-transcription comparison (Figure S9), genes that were detected in the reference condition (Maxima H- at 42 °C) but not in the stricter RT conditions were converted from Ensembl to Entrez IDs using org.Mm.eg.db and tested for over-representation of Gene Ontology Biological Process (GO BP) terms with enrichGO, using all detected genes in the comparison as the background (universe). P-values were adjusted by the Benjamini–Hochberg method; terms with adjusted *p <* 0.05 and q *<* 0.2 were considered significant. Results were summarized by fold enrichment (gene ratio/background ratio) and the significant terms per gene set were visualized.

Group comparisons used unpaired or paired two-sided t-tests or Mann-Whitney-U-tests (MWU) as stated in figure legends. Equality of variance was assessed with Levene’s test where reported. Significance levels: ns: *p >* 0.05; ∗: *p* ≤ 0.05; ∗∗: *p* ≤ 0.01; ∗∗∗: *p* ≤ 0.001; ∗∗∗∗: *p* ≤ 0.0001. Boxplots show median and interquartile range (whiskers 1.5×IQR). Diamond and whiskers show mean ± SEM. All statistical details and experimental parameters can be found in the figure legends, results, and method details.

Also, the definitions of significance levels are provided in the figure legends.

## Supplemental information

Document S1. Figures S1–S15

Table S1. Table showing the unrelated projects confirming the prime-seq2 data, related to Figure 3

Table S2. Table showing the cost calculation of prime-seq, related to STAR Methods

Table S3. Table summarizing the protocol changes from prime-seq to prime-seq2, related to STAR Methods

Table S4. Table showing the number of reads per sample and experiment, related to Figures 1–3

Table S5. Complete table of oligonucleotides used in this study, related to STAR Methods
