# Supplementary material for: Increasing usable reads in RNA-seq protocols
Source: iScience. 2026 Jul 30;29(8):116984. doi: 10.1016/j.isci.2026.116984 (PMC13452247; doi:10.1016/j.isci.2026.116984)
Supplement: Supplementary file 1 [file mmc1.pdf]

iScience, Volume *29*

Supplemental information

Increasing usable reads in RNA-seq protocols

Felix Pförtner, Eva Briem, Wolfgang Enard, and Daniel Richter

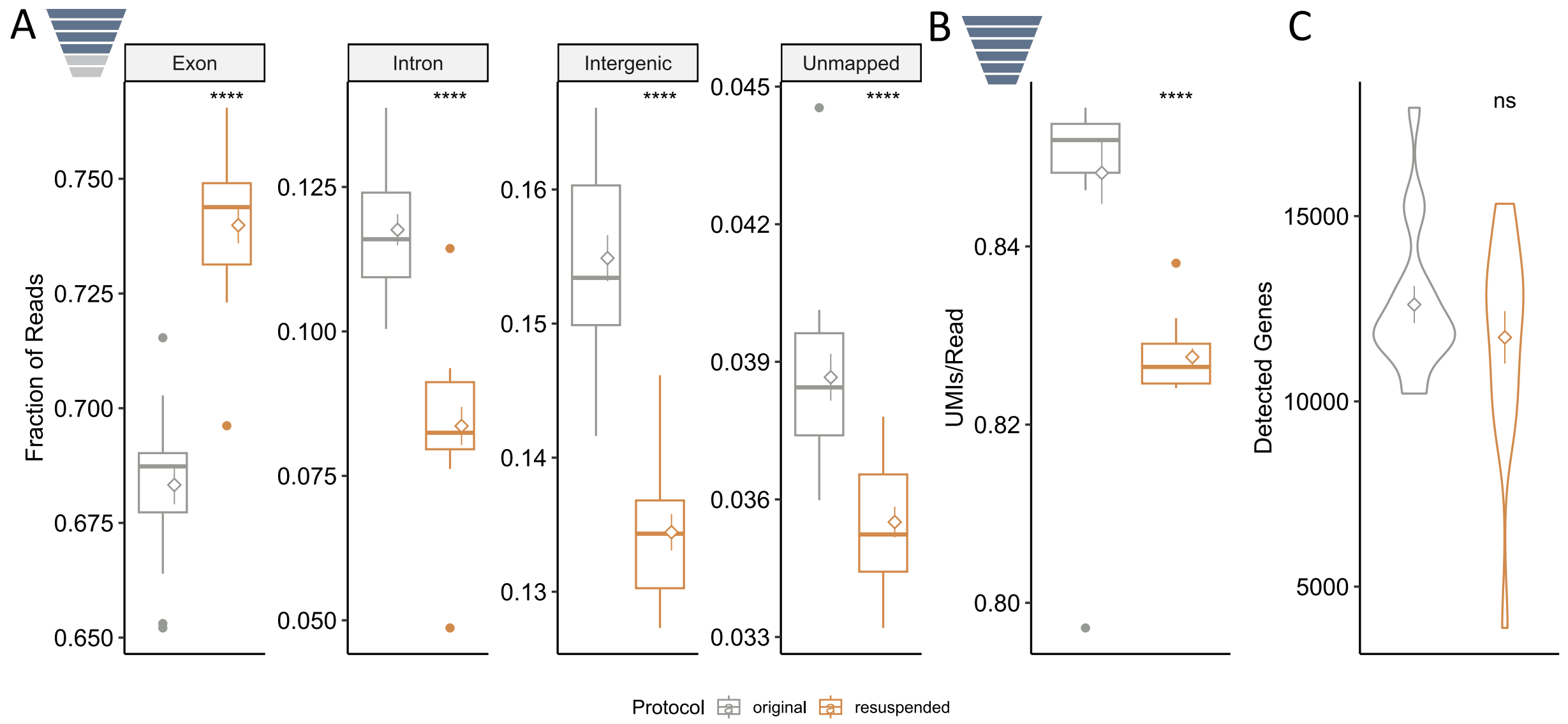

Supplemental Figure S1. Additional resuspension steps during RNA isolation, Related to Figure 1. **A**, Fraction of barcode-assigned reads mapped to exons, introns, or intergenic regions and unmapped reads. (n=16; 16 samples per condition in one library.) **B**, UMI count per read showing the loss of reads during UMI collapsing. (n=16; 16 samples per condition in one library.) **C**, Gene-level library complexity *i.e.* the number of detected genes after downsampling to 1.25 million reads per sample, showing a slight but non-significant reduction. (n=16; 16 samples per condition in one library.)
Boxplots show the median and interquartile range (whiskers: 1.5×IQR). Diamond and whiskers show mean ± SEM. P-values were calculated in unpaired two-sided t-tests (ns: $p > 0.05$; *: $p \leq 0.05$; **: $p \leq 0.01$; ***: $p \leq 0.001$; ****: $p \leq 0.0001$).

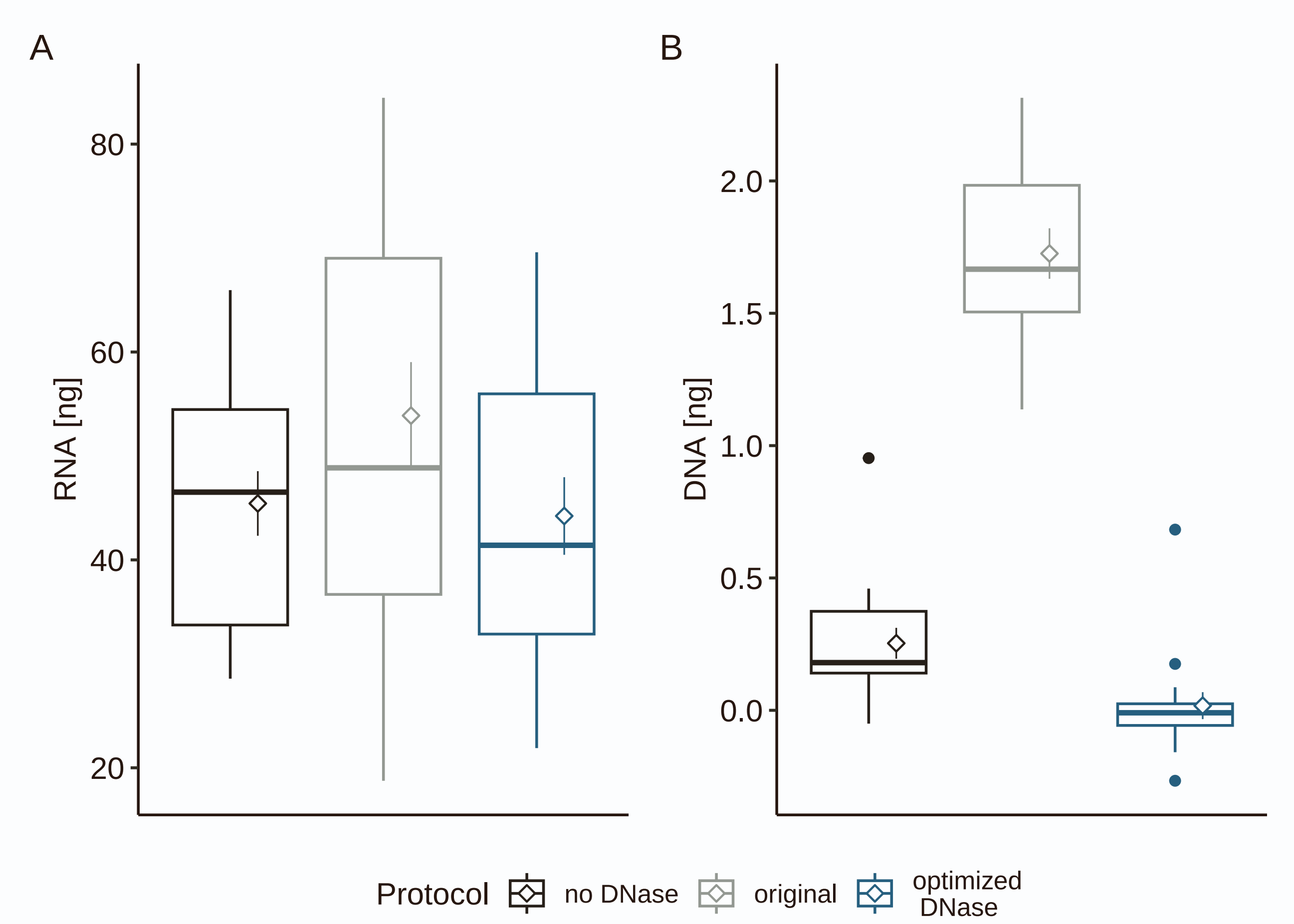

Supplemental Figure S2. More extensive plots on DNase tests, Related to Figure 1. DNA digest was compared using the original protocol and an optimized DNA digest. **A**, The plot shows RNA amount per condition after Proteinase K and DNase I treatment. (n=16; Two independent replicates of 8 samples each per condition.) **B**, Measured DNA amount after DNase digest , showing the unintended DNA removal by SPRI bead clean-up. (n=16; Two independent replicates of 8 samples each per condition.)

Boxplots show the median and interquartile range (whiskers: 1.5×IQR). Diamond and whiskers show mean ± SEM.

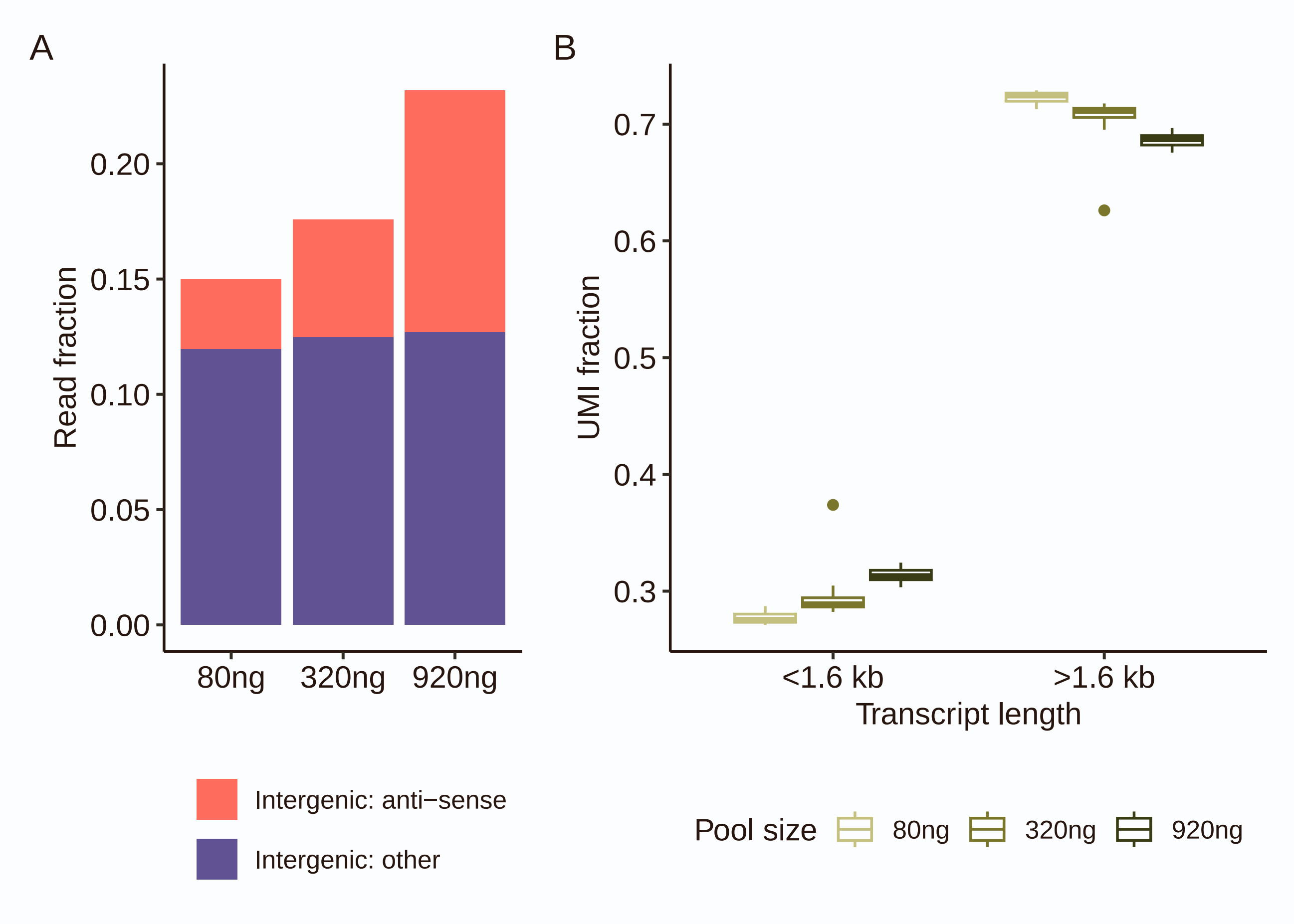

Supplemental Figure S3. cDNA pool size increases PCR length bias and antisense reads, Related to Figure 1. The effect of increasing cDNA pool size on PCR amplification bias and the generation of antisense reads was evaluated. **A**, Intergenic reads are subdivided into those mapping antisense to annotated genes and those mapping elsewhere (other). Antisense intergenic read fractions increase with greater pool size, whereas other intergenic reads remain unchanged. (n=8 for 80 ng and n=16 for 320 ng and 920 ng) **B**, UMI counts were categorized based on transcript length ($<$1.6 kb and $>$1.6 kb). Increasing pool size elevates the fraction of UMIs from shorter transcripts ($<$1.6 kb) and decreases the fraction from longer transcripts ($>$1.6 kb). (n=8 for 80 ng and n=16 for 320 ng and 920 ng).
Boxplots show the median and interquartile range (whiskers: 1.5$\times$IQR).

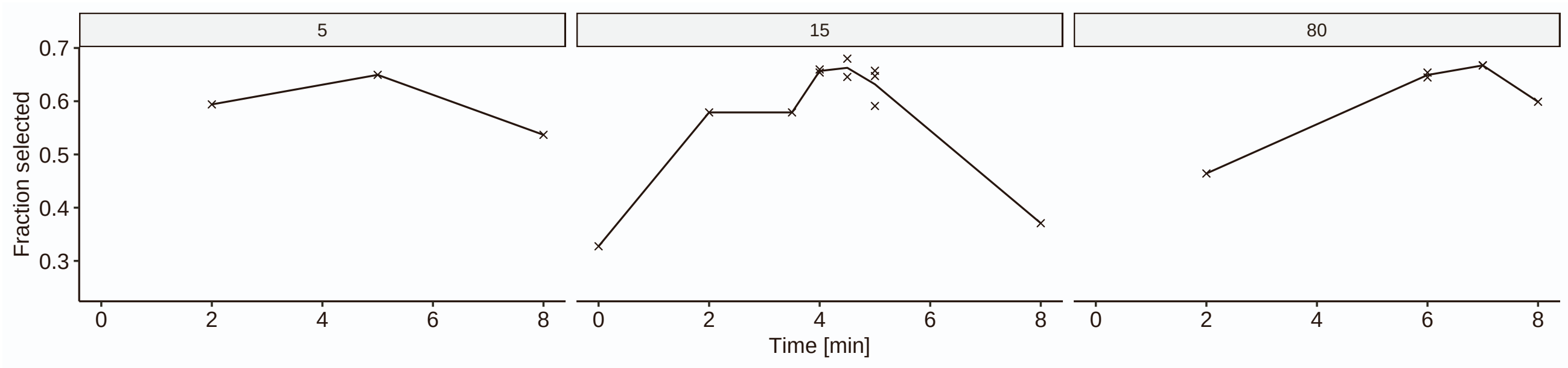

Supplemental Figure S4. Comparison of fragmentation times, Related to STAR Methods. Fraction of DNA amount in the size selection range (250-650 nt) of total DNA amount (100-8000 nt). The lines connect the mean values. (n=21 over all conditions.)

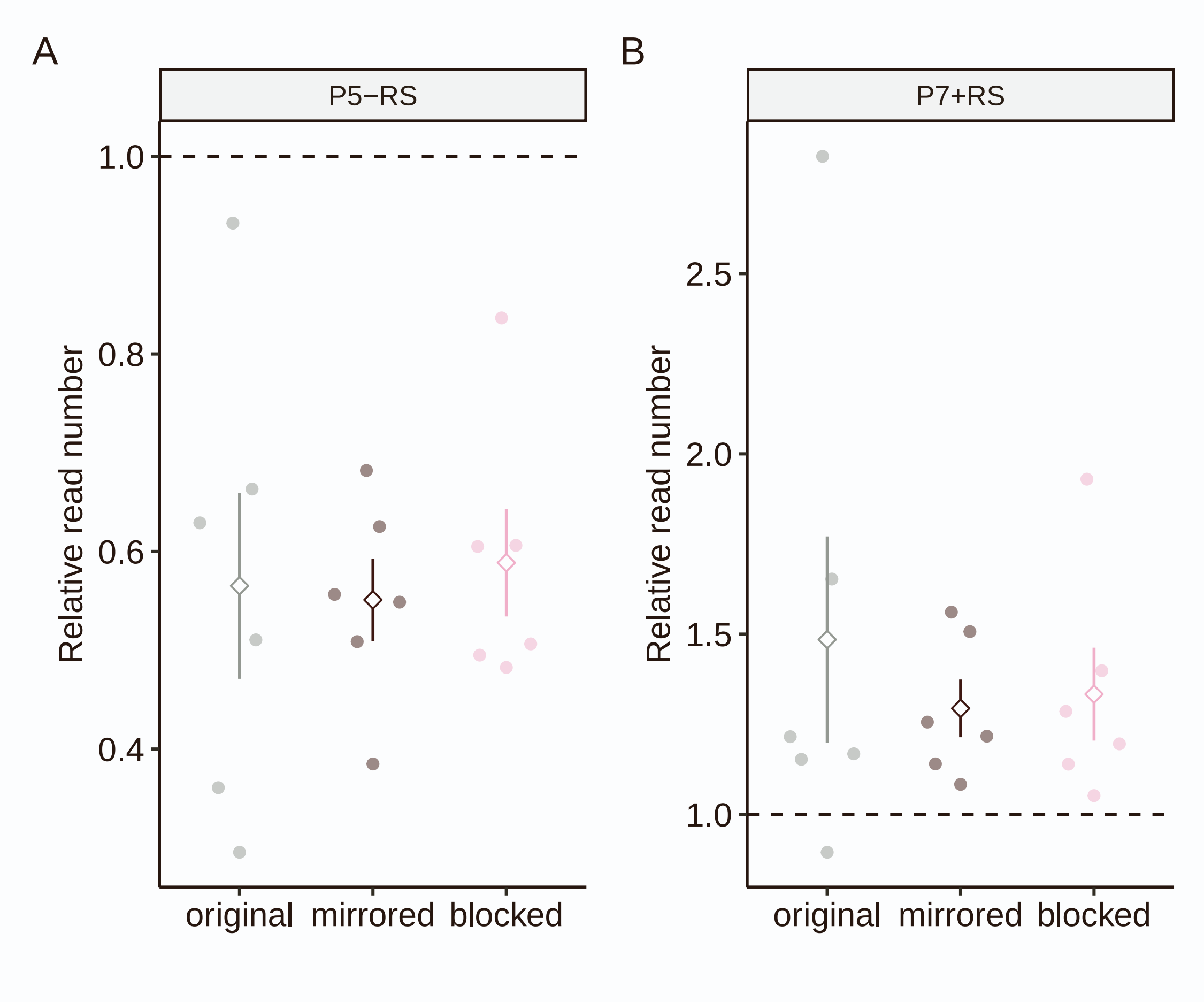

Supplemental Figure S5. Effects of index primer length on read numbers, Related to Figure 2.

A, Number of reads with the new P5-RS compared to the original P5+RS. (n=6; Six libraries per condition.) **B**, Number of reads with the new P7+RS compared to the original P7-RS. (n=6; Six libraries per condition.)
Diamond and whiskers show mean ± SEM.

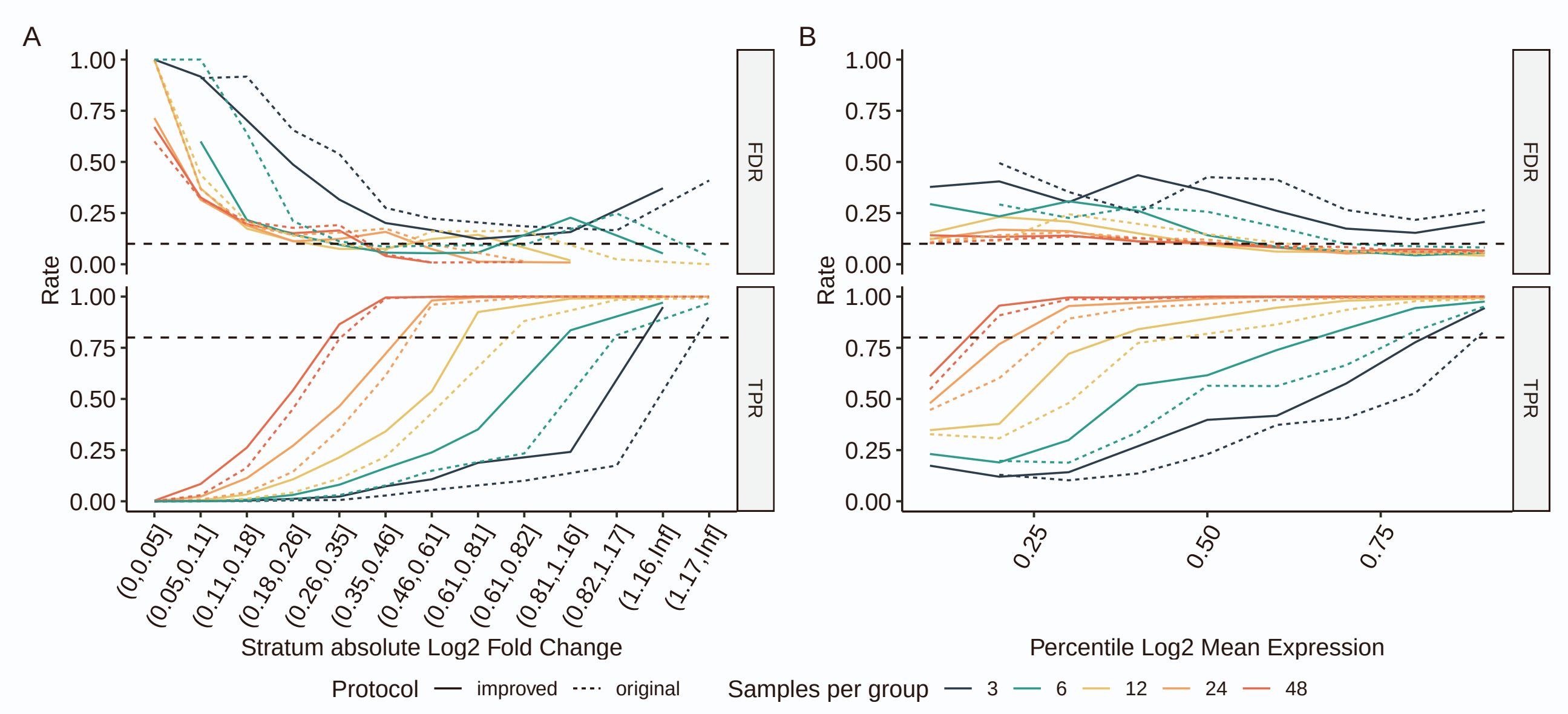

Supplemental Figure S6. Power analysis stratified by log fold change or mean expression, Related to Figure 3. Power analysis of the original and improved prime-seq protocol at equal flow cell share across replicate numbers **A**, stratified by absolute log fold change and **B**, stratified by log mean expression.

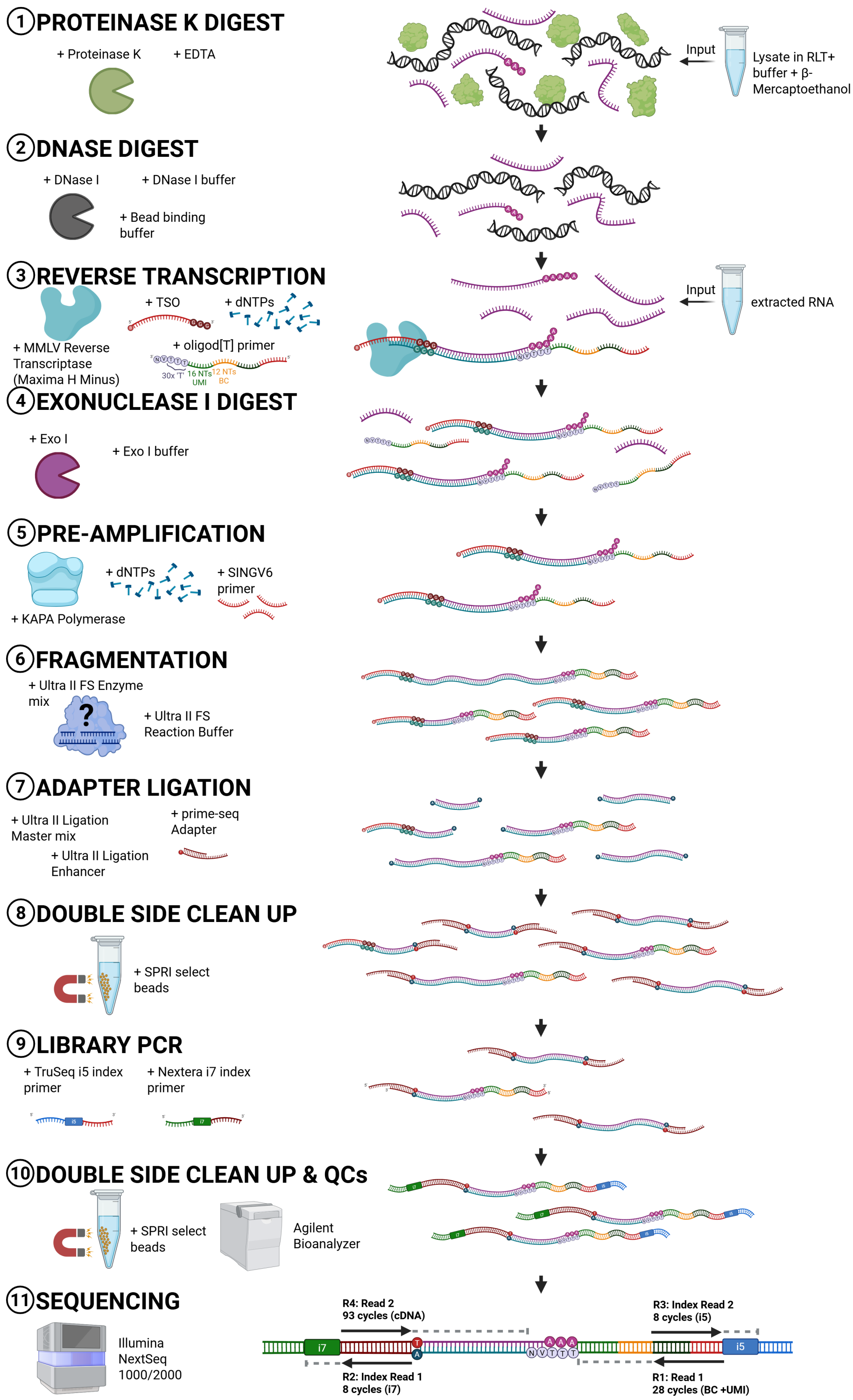

Supplemental Figure S7. prime-seq workflow, Related to Figure 1.

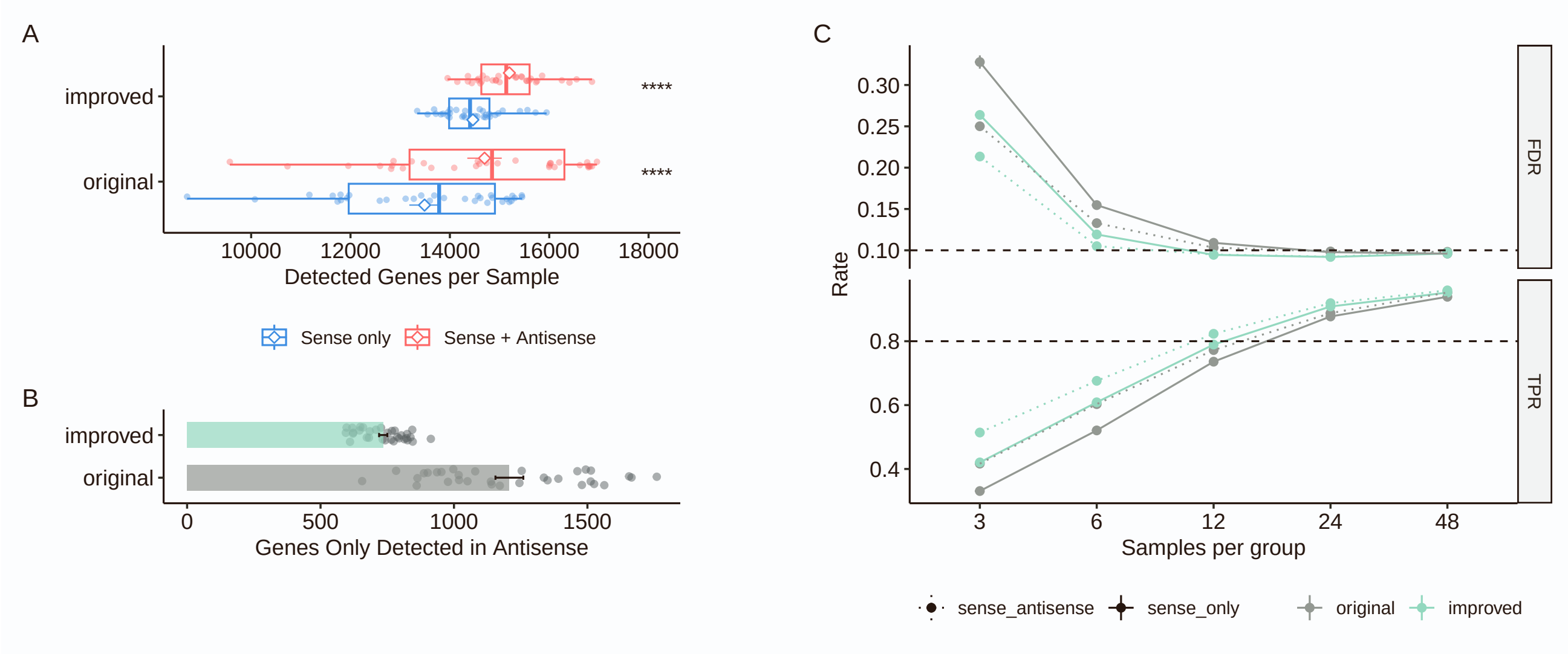

Supplemental Figure S8. Information content of antisense reads, Related to Figure 3. The information content of reads mapping antisense of annotated genes was evaluated for this protocol to investigate whether they contain useful biological information or predominantly add noise. Intergenic reads were recounted using zUMIs [S1] with reversed strandedness to capture reads that map antisense of annotated gene features. New UMI count matrices were constructed by adding antisense-derived UMI counts to standard sense-derived UMI counts. **A**, Including antisense reads increased the number of detected genes per sample in the original and the improved protocol, with a stronger effect on the original protocol. (n=32; Four libraries of 8 barcodes per protocol.) **B**, The number of genes detected only using antisense reads per sample is higher in the original protocol. (n=32; Four libraries of 8 barcodes per protocol. Bars show mean, error bars show SEM) **C**, Power analysis using powsimR [S2] was run as before, now also with the sense + antisense UMI count matrix. The sense + antisense matrix was subset to genes that also contain counts in the sense matrix. Across protocols and replicate numbers TPR is increased when adding antisense reads and FDR is reduced (Points and lines show means; error bars show SEM).

Boxplots show the median and interquartile range (whiskers: 1.5×IQR). Diamond and whiskers show mean ± SEM. P-values were calculated in unpaired two-sided t-tests (ns: $p > 0.05$; *: $p \leq 0.05$; **: $p \leq 0.01$; ***: $p \leq 0.001$; ****: $p \leq 0.0001$).

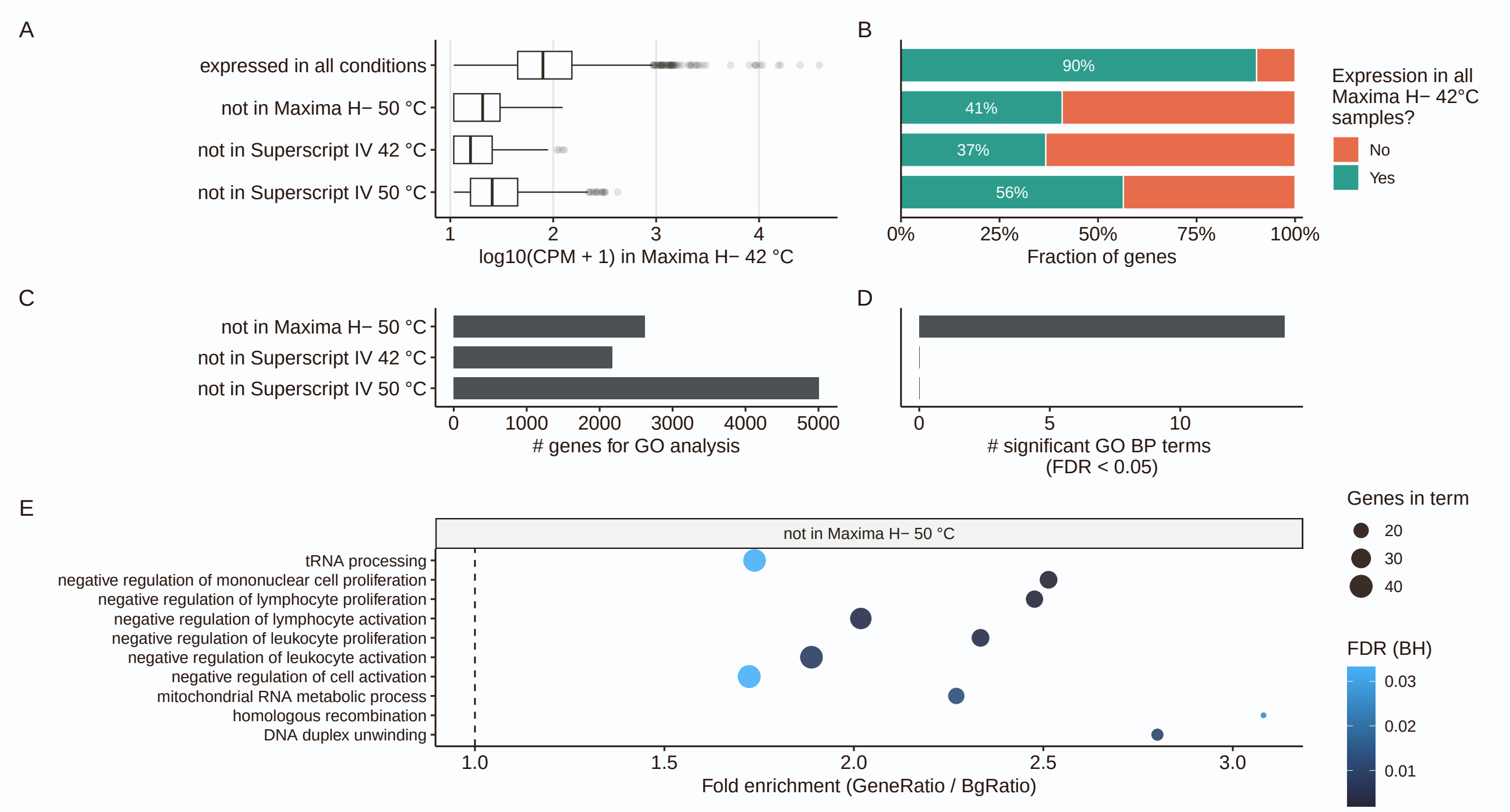

Supplemental Figure S9. Investigation of gene detection across reverse transcription conditions, Related to Figure 1. Genes that were detected in the initial Maxima 42°C condition but not after changing RT conditions were investigated. Library depth was equalized by UMI downsampling. Genes were categorized by being detected in all conditions (in ≥ 2 samples in all conditions), or in Maxima 42 °C (in ≥ 2 samples of Maxima 42°C), but not in others (in ≤ 1 sample in other conditions). **A**, Genes that are not present after changing RT conditions show lower logCPM in the initial conditions compared to genes that are expressed in all conditions. **B**, Fraction of genes per group that are expressed in all Maxima 42°C samples. Genes that are not present after changing RT conditions are less reproducibly expressed than genes that are expressed in all conditions. **C**, Number of genes used for Gene Ontology (GO) analysis. **D**, Number of significant GO terms using the Biological Process (BP) ontology. **E**, Fold enrichment of significant GO terms.

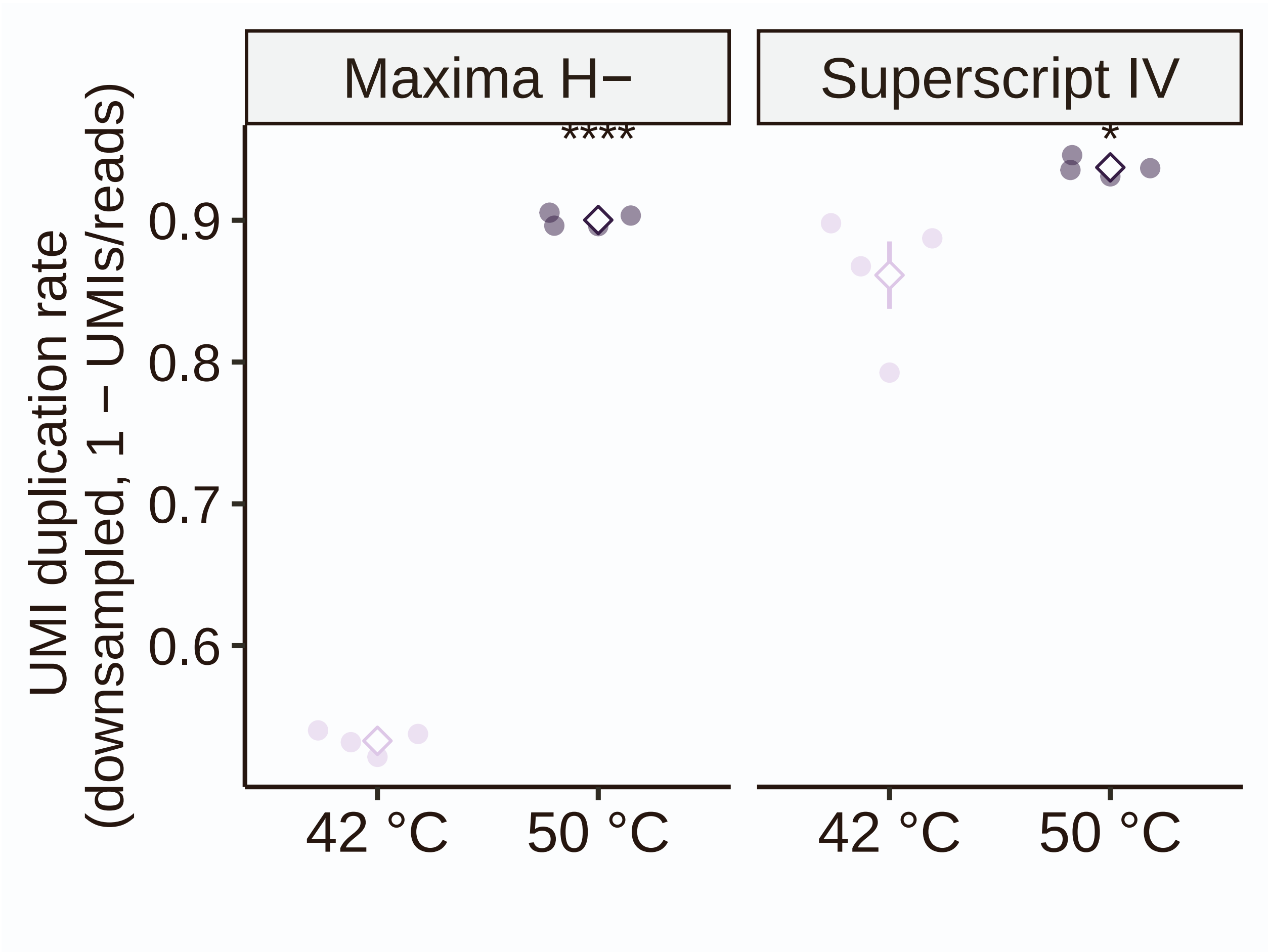

Supplemental Figure S10. UMI duplication rate across RT conditions, Related to Figure 1. The UMI duplication rate ($1 - \frac{uniqueUMIs}{reads}$) was calculated after downsampling bam files to equal reads per sample. (n=4 per condition; Diamond and whiskers show mean ± SEM).
P-values were calculated in unpaired two-sided t-tests comparing 50°C to 42°C (ns: $p > 0.05$; *: $p \leq 0.05$; **: $p \leq 0.01$; ***: $p \leq 0.001$; ****: $p \leq 0.0001$).

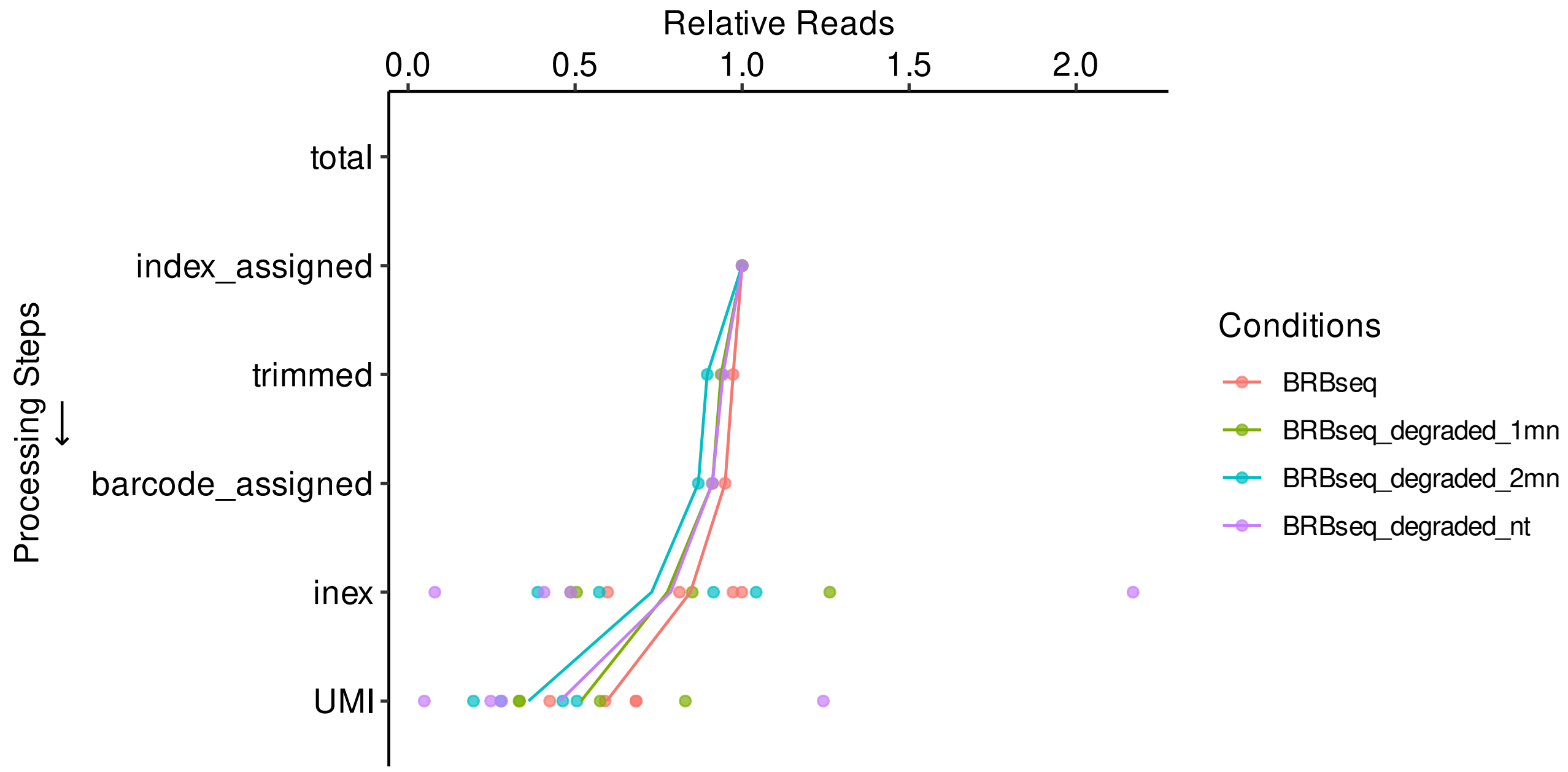

Supplemental Figure S11. BRB-seq funnel, Related to Figure 3. Funnel plot for adipose tissue–derived mesenchymal stem cell sample processed with BRB-seq from Alpern et al. [S3]. Degraded samples were chemically fragmented to test low quality RNA. Only index-assigned read data were published. Consequently, earlier "funnel" steps such as reads per flowcell or the fraction of mapped index-assigned reads cannot be evaluated. Because these steps account for some of the largest gains from prime-seq to prime-seq2, additional improvements relative to BRB-seq are plausible but cannot be assessed with the available data. Alpern et al. [S3] report BRB-seq reagent costs of $2.4 per sample (96-sample library), excluding RNA isolation. Notably, their estimate omits consumables. Using a comparable basis, starting from RNA and excluding consumables, prime-seq2 costs $1.3 per sample (Tab. S2).

Points show values per library (index-assigned, trimmed, barcode-assigned) or per barcode (exonic/intronic, UMI). Lines connect means per condition.

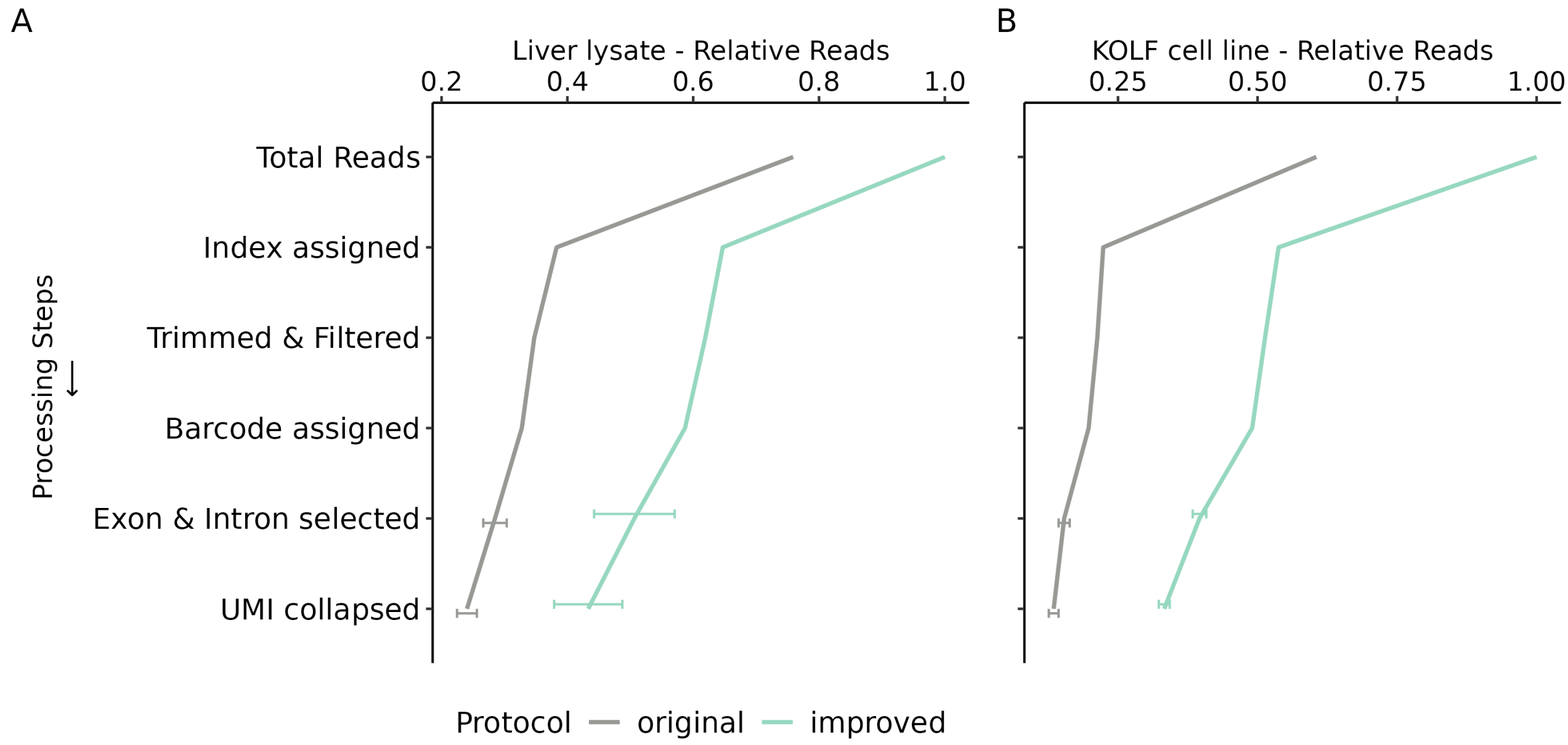

Supplemental Figure S12. Alternative samples tested with the original and improved prime-seq protocol, Related to Figure 3. Funnel plot for two additional samples processed with the original and improved prime-seq protocol showing the read number at each step relative to the total reads with the improved protocol. The first four steps use one data point per library, while the last two show one data point per sample, increasing the variation. Lines show means; error bars show SEM. (n=32, Two libraries of 32 samples (8 barcodes) per condition.) **A**, Mouse liver lysates. **B**, Human iPS cell lysate.

Lines connect means per condition.

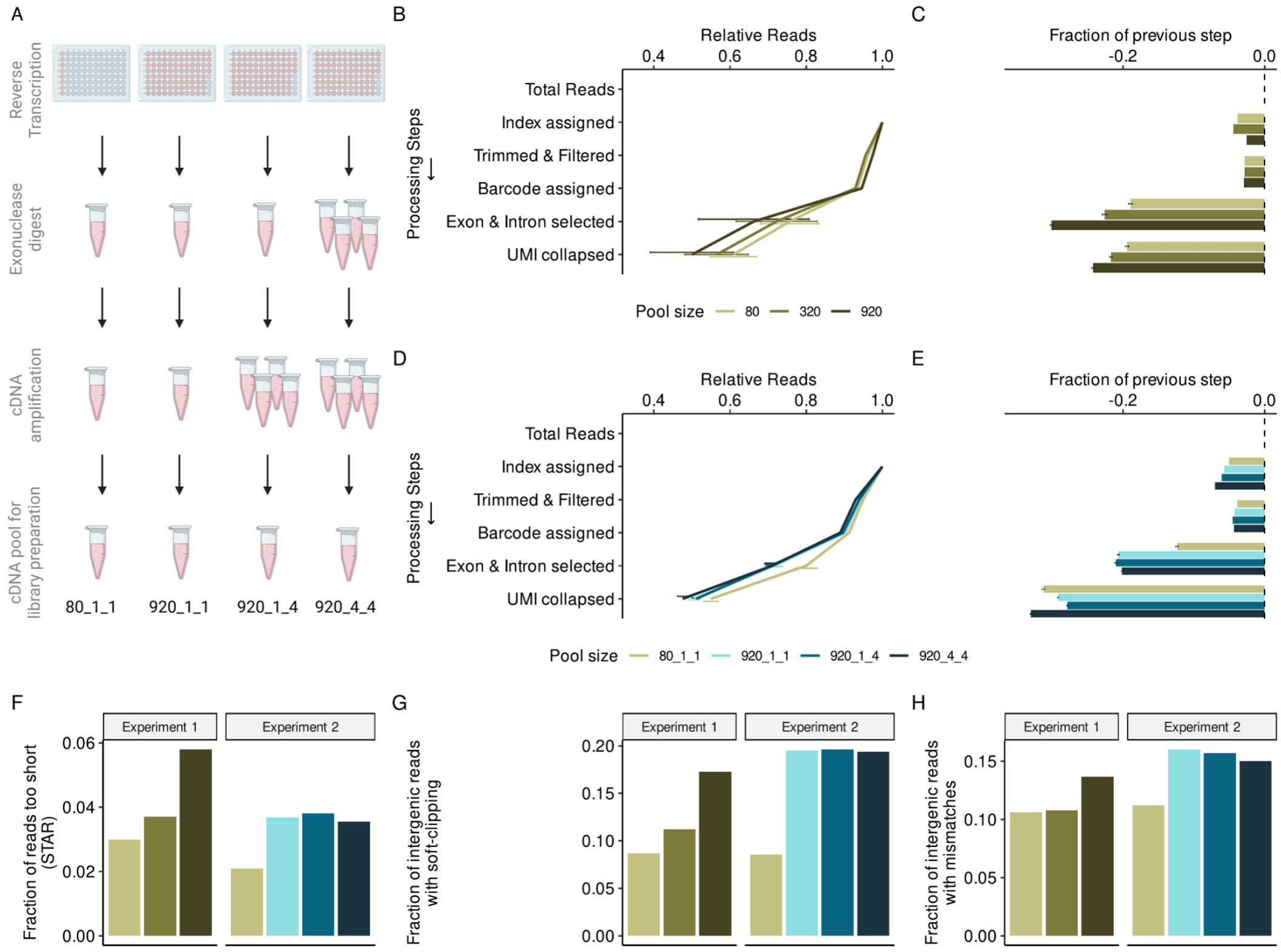

Supplemental Figure S13. Effects of sample pooling, Related to Figure 1. In experiment 1, samples of 10 ng of total RNA each were reverse transcribed, pooled, exonuclease digested, and then added to the cDNA amplification PCR in amounts corresponding to 8 (80 ng), 32 (320 ng), and 92 (920 ng) samples (total RNA), respectively. This was done in a total of 40 replicates (n=8 for 80 ng and n=16 for 320 ng and 920 ng) from which one library per condition was generated. **A**, Schematic describing the experimental setup of experiment 2. Experiment 2 investigated whether delaying pooling until cDNA amplification reduces the pooling effects seen in experiment 1. The 80 ng samples were treated as before. For 920 ng, samples were kept in 4 pools of 230 ng until pooling them in different protocol steps. Samples were pooled either directly as before after RT (920_1_1), after exonuclease digest (920_4_1), or after cDNA amplification (920_4_4). This was done in a total of 56 replicates (n=8 for 80 ng and n=16 for each 920 ng condition) from which one library per condition was generated. **B**, Funnel plot of experiment 1 libraries showing the read number at each step relative to the index assigned reads. The first four steps use one data point per library, while the last two show one data point per sample. **C**, Change in total reads per step relative to the previous step in experiment 1. **D**, Funnel plot of experiment 2 libraries showing the read number at each step relative to the index assigned reads. The first four steps use one data point per library, while the last two show one data point per sample. **E**, Change in total reads per step relative to the previous step in experiment 2. **F**, Fraction of trimmed & filtered reads that could not be mapped successfully because the fraction of aligned bases is less than 0.66 (default –outFilterMatchNminOverLread 0.66) **G**, Fraction of intergenic reads that were soft-clipped during STAR alignment [S4]. **H**, Fraction of intergenic reads with mismatches added during STAR alignment [S4].

Lines and bars show means; error bars show SEM.

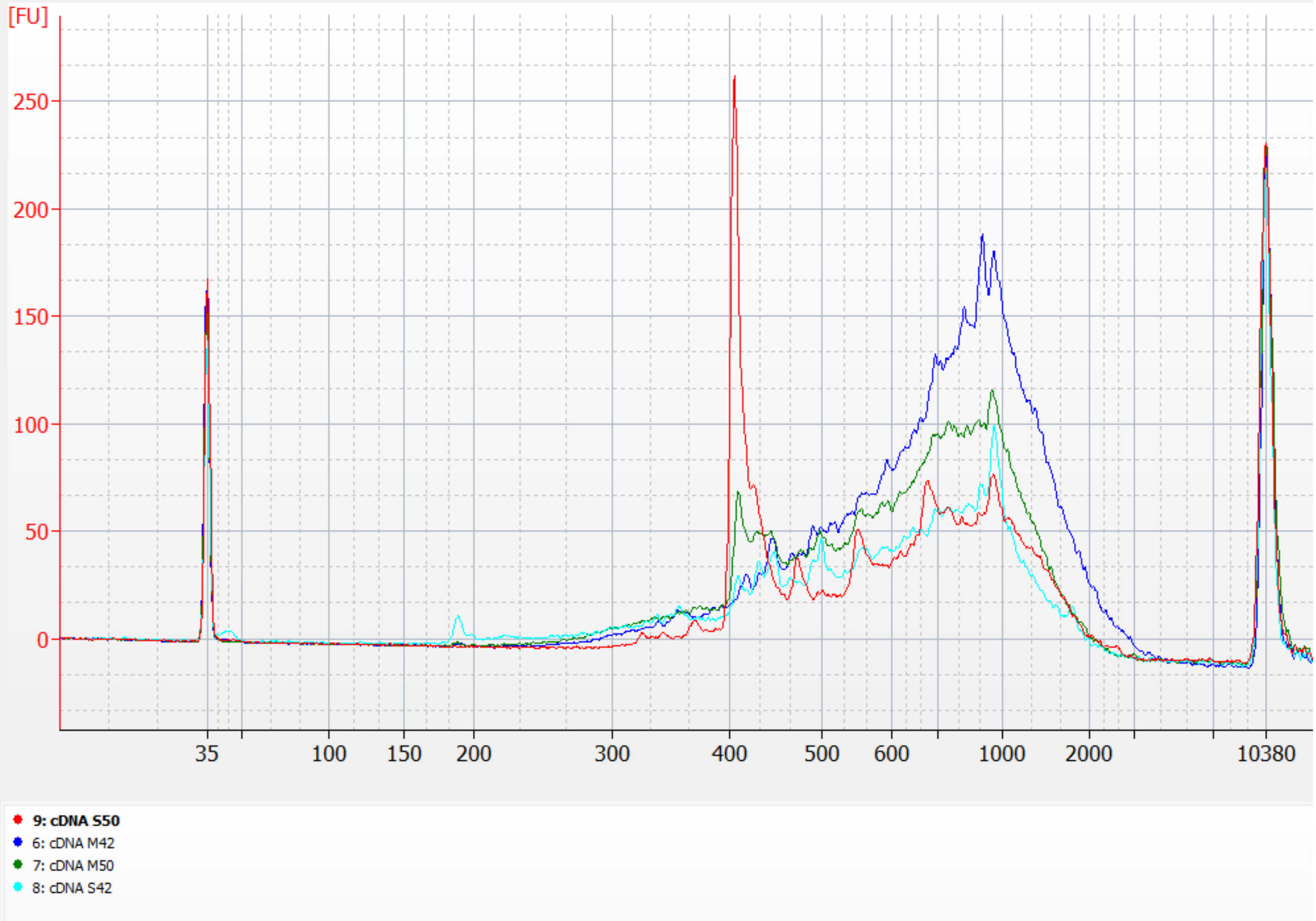

Supplemental Figure S14. cDNA Bioanalyzer traces of RT - enzyme and temperature tests, Related to Figure 1. Overall length distributions were largely similar. We observed an additional peak at 400–450 bp in libraries generated at higher RT temperatures, whereas changing the RT enzyme did not lead to marked differences. **red** SuperScript IV at 50 °C, **cyan** SuperScript IV at 42 °C, **green** Maxima H- at 50 °C, **blue** Maxima H- at 42 °C.

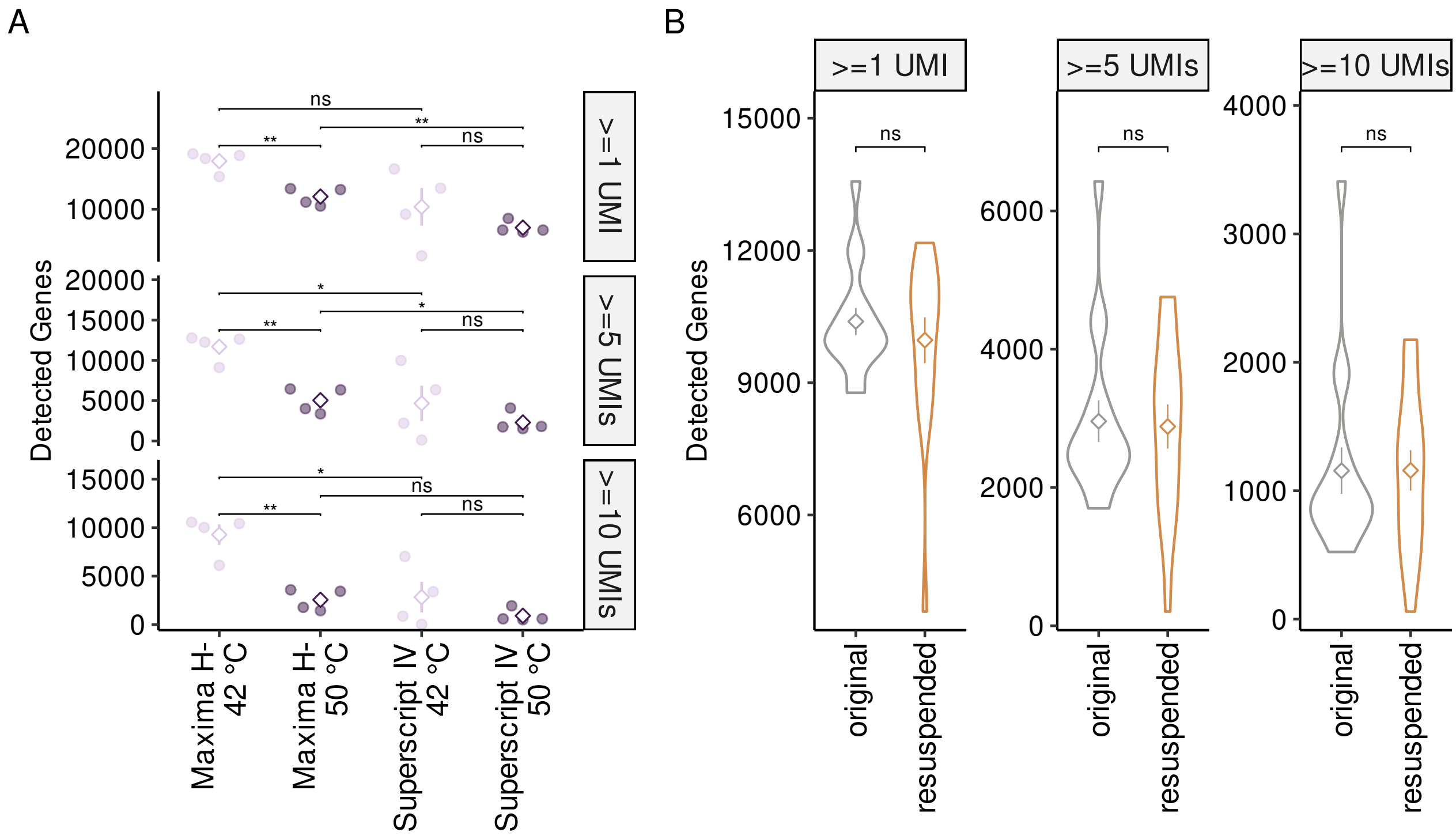

Supplemental Figure S15. Gene-level library complexity trends are similar across thresholds, Related to Figure 1.

A, The number of genes with at least 1 (as in Fig. 1F), 5 or 10 unique UMIs after downsampling to 7 million reads per sample (n=4 per condition). **B**, The number of genes with at least 1 (as in Fig. S1C), 5 or 10 unique UMIs after downsampling to 1.25 million reads per sample (n=16; 16 samples per condition in one library.)
Diamond and whiskers show mean ± SEM. P-values were calculated in unpaired two-sided t-tests (ns: $p > 0.05$; *: $p \leq 0.05$; **: $p \leq 0.01$; ***: $p \leq 0.001$; ****: $p \leq 0.0001$).

Supplemental References

[S1] Parekh S., Ziegenhain C., Vieth B., Enard W., Hellmann I. zUMIs: a fast and flexible pipeline to process RNA sequencing data with UMIs. *GigaScience*. 2018;7:giy059. doi: 10.1093/gigascience/giy059.

[S2] Vieth B., Ziegenhain C., Parekh S., Enard W., Hellmann I. powsimR: power analysis for bulk and single cell RNA-seq experiments. *Bioinformatics*. 2017;33:3486–3488. doi: 10.1093/bioinformatics/btx435.

[S3] Alpern D., Gardeux V., Russeil J., Mangeat B., Meireles-Filho A.C.A., Breysse R., Hacker D., Deplancke B. BRB-seq: ultra-affordable high-throughput transcriptomics enabled by bulk RNA barcoding and sequencing. *Genome Biology*. 2019;20:71. Genome Biology. 2019;20:71. doi: 10.1186/s13059-019-1671-x.

[S4] Dobin A., Davis C.A., Schlesinger F., Drenkow J., Zaleski C., Jha S., Batut P., Chaisson M., Gingeras T.R. STAR: ultrafast universal RNA-seq aligner. *Bioinformatics*. 2013;29:15–21. doi: 10.1093/bioinformatics/bts635.
